# Association of Whole-Genome and NETRIN1 Signaling Pathway–Derived Polygenic Risk Scores for Major Depressive Disorder and White Matter Microstructure in the UK Biobank

**DOI:** 10.1016/j.bpsc.2018.07.006

**Published:** 2019-01

**Authors:** Miruna C. Barbu, Yanni Zeng, Xueyi Shen, Simon R. Cox, Toni-Kim Clarke, Jude Gibson, Mark J. Adams, Mandy Johnstone, Chris S. Haley, Stephen M. Lawrie, Ian J. Deary, Major Depressive Disorder Working Group of the Psychiatric Genomics Consortium, Naomi R. Wray, Stephan Ripke, Manuel Mattheisen, Maciej Trzaskowski, Enda M. Byrne, Abdel Abdellaoui, Mark J. Adams, Esben Agerbo, Tracy M. Air, Till F.M. Andlauer, Silviu-Alin Bacanu, Marie Bækvad-Hansen, Aartjan T.F. Beekman, Tim B. Bigdeli, Elisabeth B. Binder, Douglas H.R. Blackwood, Julien Bryois, Henriette N. Buttenschøn, Jonas Bybjerg-Grauholm, Na Cai, Enrique Castelao, Jane Hvarregaard Christensen, Toni-Kim Clarke, Jonathan R.I. Coleman, Lucía Colodro-Conde, Baptiste Couvy-Duchesne, Nick Craddock, Gregory E. Crawford, Gail Davies, Ian J. Deary, Franziska Degenhardt, Eske M. Derks, Nese Direk, Conor V. Dolan, Erin C. Dunn, Thalia C. Eley, Valentina Escott-Price, Farnush Farhadi Hassan Kiadeh, Hilary K. Finucane, Andreas J. Forstner, Josef Frank, Héléna A. Gaspar, Michael Gill, Fernando S. Goes, Scott D. Gordon, Jakob Grove, Lynsey S. Hall, Christine Søholm Hansen, Thomas F. Hansen, Stefan Herms, Ian B. Hickie, Per Hoffmann, Georg Homuth, Carsten Horn, Jouke-Jan Hottenga, David M. Hougaard, Marcus Ising, Rick Jansen, Eric Jorgenson, James A. Knowles, Isaac S. Kohane, Julia Kraft, Warren W. Kretzschmar, Jesper Krogh, Zoltán Kutalik, Yihan Li, Penelope A. Lind, Donald J. MacIntyre, Dean F. MacKinnon, Robert M. Maier, Wolfgang Maier, Jonathan Marchini, Hamdi Mbarek, Patrick McGrath, Peter McGuffin, Sarah E. Medland, Divya Mehta, Christel M. Middeldorp, Evelin Mihailov, Yuri Milaneschi, Lili Milani, Francis M. Mondimore, Grant W. Montgomery, Sara Mostafavi, Niamh Mullins, Matthias Nauck, Bernard Ng, Michel G. Nivard, Dale R. Nyholt, Paul F. O'Reilly, Hogni Oskarsson, Michael J. Owen, Jodie N. Painter, Carsten Bøcker Pedersen, Marianne Giørtz Pedersen, Roseann E. Peterson, Erik Pettersson, Wouter J. Peyrot, Giorgio Pistis, Danielle Posthuma, Jorge A. Quiroz, Per Qvist, John P. Rice, Brien P. Riley, Margarita Rivera, Saira Saeed Mirza, Robert Schoevers, Eva C. Schulte, Ling Shen, Jianxin Shi, Stanley I. Shyn, Engilbert Sigurdsson, Grant C.B. Sinnamon, Johannes H. Smit, Daniel J. Smith, Hreinn Stefansson, Stacy Steinberg, Fabian Streit, Jana Strohmaier, Katherine E. Tansey, Henning Teismann, Alexander Teumer, Wesley Thompson, Pippa A. Thomson, Thorgeir E. Thorgeirsson, Matthew Traylor, Jens Treutlein, Vassily Trubetskoy, André G. Uitterlinden, Daniel Umbricht, Sandra Van der Auwera, Albert M. van Hemert, Alexander Viktorin, Peter M. Visscher, Yunpeng Wang, Bradley T. Webb, Shantel Marie Weinsheimer, Jürgen Wellmann, Gonneke Willemsen, Stephanie H. Witt, Yang Wu, Hualin S. Xi, Jian Yang, Futao Zhang, Volker Arolt, Bernhard T. Baune, Klaus Berger, Dorret I. Boomsma, Sven Cichon, Udo Dannlowski, E.J.C. de Geus, J. Raymond DePaulo, Enrico Domenici, Katharina Domschke, Tõnu Esko, Hans J. Grabe, Steven P. Hamilton, Caroline Hayward, Andrew C. Heath, Kenneth S. Kendler, Stefan Kloiber, Glyn Lewis, Qingqin S. Li, Susanne Lucae, Pamela A.F. Madden, Patrik K. Magnusson, Nicholas G. Martin, Andrew M. McIntosh, Andres Metspalu, Ole Mors, Preben Bo Mortensen, Bertram Müller-Myhsok, Merete Nordentoft, Markus M. Nöthen, Michael C. O'Donovan, Sara A. Paciga, Nancy L. Pedersen, Brenda W.J.H. Penninx, Roy H. Perlis, David J. Porteous, James B. Potash, Martin Preisig, Marcella Rietschel, Catherine Schaefer, Thomas G. Schulze, Jordan W. Smoller, Kari Stefansson, Henning Tiemeier, Rudolf Uher, Henry Völzke, Myrna M. Weissman, Thomas Werge, Cathryn M. Lewis, Douglas F. Levinson, Gerome Breen, Anders D. Børglum, Patrick F. Sullivan, 23andMe Research Team, Michelle Agee, Babak Alipanahi, Adam Auton, Robert K. Bell, Katarzyna Bryc, Sarah L. Elson, Pierre Fontanillas, Nicholas A. Furlotte, David A. Hinds, Karen E. Huber, Aaron Kleinman, Nadia K. Litterman, Jennifer C. McCreight, Matthew H. McIntyre, Joanna L. Mountain, Elizabeth S. Noblin, Carrie A.M. Northover, Steven J. Pitts, J. Fah Sathirapongsasuti, Olga V. Sazonova, Janie F. Shelton, Suyash Shringarpure, Chao Tian, Joyce Y. Tung, Vladimir Vacic, Catherine H. Wilson, Andrew M. McIntosh, Heather C. Whalley

**Affiliations:** a Division of Psychiatry, Centre for Clinical Brain Sciences, University of Edinburgh, Edinburgh, Scotland; b Medical Research Council, Human Genetics Unit, University of Edinburgh, Edinburgh, Scotland; c Institute of Genetics and Molecular Medicine, University of Edinburgh, Edinburgh, Scotland; d Centre for Cognitive Ageing and Cognitive Epidemiology, Department of Psychology, University of Edinburgh, Edinburgh, Scotland; e Major Depression Disorder Working Group of the Psychiatric Genomics Consortium; f 23andMe, Inc., Mountain View, California

**Keywords:** Biological pathway, Major depressive disorder, NETRIN1, Polygenic risk score, Thalamic radiations, White matter

## Abstract

**Background:**

Major depressive disorder is a clinically heterogeneous psychiatric disorder with a polygenic architecture. Genome-wide association studies have identified a number of risk-associated variants across the genome and have reported growing evidence of NETRIN1 pathway involvement. Stratifying disease risk by genetic variation within the NETRIN1 pathway may provide important routes for identification of disease mechanisms by focusing on a specific process, excluding heterogeneous risk-associated variation in other pathways. Here, we sought to investigate whether major depressive disorder polygenic risk scores derived from the NETRIN1 signaling pathway (NETRIN1-PRSs) and the whole genome, excluding NETRIN1 pathway genes (genomic-PRSs), were associated with white matter microstructure.

**Methods:**

We used two diffusion tensor imaging measures, fractional anisotropy (FA) and mean diffusivity (MD), in the most up-to-date UK Biobank neuroimaging data release (FA: *n* = 6401; MD: *n* = 6390).

**Results:**

We found significantly lower FA in the superior longitudinal fasciculus (β = −.035, *p*_corrected_ = .029) and significantly higher MD in a global measure of thalamic radiations (β = .029, *p*_corrected_ = .021), as well as higher MD in the superior (β = .034, *p*_corrected_ = .039) and inferior (β = .029, *p*_corrected_ = .043) longitudinal fasciculus and in the anterior (β = .025, *p*_corrected_ = .046) and superior (β = .027, *p*_corrected_ = .043) thalamic radiation associated with NETRIN1-PRS. Genomic-PRS was also associated with lower FA and higher MD in several tracts.

**Conclusions:**

Our findings indicate that variation in the NETRIN1 signaling pathway may confer risk for major depressive disorder through effects on a number of white matter tracts.

Major depressive disorder (MDD) is a common and frequently disabling psychiatric disorder and a leading cause of disability worldwide (1). MDD is known to result from a complex combination of environmental and genetic factors 2, 3, and it has a moderate heritability, of approximately 37% 4, 5, 6.

Genome-wide association studies (GWASs) suggest that at least part of MDD’s heritability is due to the cumulative effect of alleles of small effect size 7, 8, and they have identified a number of risk-associated genetic variants across the genome 6, 7, 9, 10, 11. Significant findings for GWAS analyses can also be annotated to specific biological pathways, revealing underlying cellular and molecular mechanisms.

Following several GWASs, the Psychiatric Genomics Consortium has identified an aggregation of variants in several specific biological pathways 12, 13. In MDD, Zeng *et al.* (14) combined pathway and regional heritability analyses in two independent samples and reported that the NETRIN1 signaling pathway was involved in the genetic etiology of MDD. Moreover, polygenic risk scores (PRSs) calculated for this pathway alone more accurately predicted MDD in one of the cohorts compared with PRSs calculated for the whole genome. Genetic variation within the NETRIN1 signaling pathway may therefore capture more etiologically circumscribed liability for MDD that is less susceptible to heterogeneous influences from other biological pathways.

Animal studies have previously indicated that NETRIN1, by binding to and activating NETRIN1 receptors such as deleted in colorectal cancer (DCC), plays an important role in commissural and cortical axon guidance (15). More recently, DCC was identified as playing a crucial role in thalamic axonal growth, confirming that interaction of NETRIN1 with DCC leads to successful axon growth during central nervous system development (16). GWASs of other traits related to MDD have also shown an aggregation of variants in the NETRIN1 pathway 17, 18.

Previous studies have attempted to investigate psychiatric disorders by examining relevant quantitative traits such as brain structure or function (19). Differences in white matter integrity as measured by diffusion tensor imaging have been found between MDD patients and healthy participants in numerous studies, although findings have been widely inconsistent 20, 21, 22. For example, Shen *et al.* (20) found significantly lower global white matter integrity in association fibers and thalamic radiations, as measured by fractional anisotropy (FA), in MDD patients compared with that in healthy individuals. More specifically, they also found lower FA in the left superior longitudinal fasciculus, superior thalamic radiations, and forceps major tracts in MDD patients. Lower white matter integrity as measured by FA has also been found in adolescents with MDD compared with that in age-matched healthy individuals 21, 22.

It has previously been shown that the NETRIN1 signaling pathway is associated with MDD and white matter microstructure (14). Therefore, in the current study, we sought to investigate the association between MDD risk–associated variants in the NETRIN1 signaling pathway and white matter integrity. We first created PRSs for pathway single-nucleotide polymorphisms (SNPs) (NETRIN1-PRSs) and SNPs excluded from the pathway (genomic-PRSs). We then tested their association with white matter integrity as measured by FA and mean diffusivity (MD). We used the most up-to-date genetic and imaging data available from the UK Biobank (UKB). We hypothesized that NETRIN1-PRS would be significantly associated with white matter integrity, after adjustment for genomic-PRS, indicating a potential role of the pathway in MDD pathophysiology.

## Methods and Materials

### UK Biobank

The UKB study consisted of 502,617 community-dwelling ndividuals who were recruited between 2006 and 2010 in the United Kingdom (http://biobank.ctsu.ox.ac.uk/crystal/field.cgi?id=200). The UKB received ethical approval from the Research Ethics Committee (reference: 11/NW/0382). This study has been approved by the UKB Access Committee (Project No. 4844). Written informed consent was obtained from all participants.

### Study Population

In the most recent UKB imaging data release, 8839 individuals (*n*_female_ = 4639; *n*_male_ = 4200; mean age: 62.54 ± 7.42 years; age range: 45.17–79.33 years) completed a diffusion tensor imaging assessment and a quality check by the UKB. In addition to this, for the current study, individuals were excluded if they participated in studies from the Psychiatric Genomics Consortium MDD GWAS (23) or Generation Scotland (Scottish Family Health Study), or if the individuals were biologically related to another participant, as the Psychiatric Genomics Consortium MDD GWAS data set was used in order to calculate PRS. Moreover, individuals whose FA and MD values were >3 SDs above or below the mean were not included in the study (Supplemental Tables S4 and S5). This resulted in the inclusion of 6401 individuals with FA values (*n*_female_ = 3334; *n*_male_ = 3067; mean age: 62.60 ± 7.37 years; age range: 45.92–78.42 years; *n*_control_ = 3736; *n*_case_ = 2512) and 6390 individuals with MD values (*n*_female_ = 3327; *n*_male_ = 3063; mean age: 62.58 ± 7.36 years; age range: 45.92–78.42 years; *n*_control_ = 3729; *n*_case_ = 2508) and the exclusion of 19 and 30 individuals with FA and MD values from a total sample of 6420, respectively. Details of data exclusion as well as participant information for the full data set (*N* = 6420) are shown in Supplemental Tables S1 and S2.

### SNP Annotation

Genic SNPs found in the NETRIN1 signaling pathway as taken from Zeng *et al.*’s 2017 study (14) (*N*_genes_ = 43; gene list is presented in the Supplemental Table S3), and genic SNPs excluded from the pathway were annotated using the program ANNOVAR. ANNOVAR is a biostatistical tool used to annotate genetic variants to functional genomic regions (24). In the current study, we performed a gene-based annotation for SNPs used in the largest available GWAS of MDD (*N* = 461,134 cases, of which 130,664 were MDD cases), carried out by the Psychiatric Genomics Consortium (23), which includes summary statistics from the personal genetics company 23andMe, Inc. (10). We defined gene boundaries as an extended region of 20 kb from transcription start sites and transcription end sites. After SNPs were annotated to genes, they were further mapped to the NETRIN1 signaling pathway. All protein-coding genes within this file were annotated in reference to hg 19. Intergenic SNPs were not included in the annotated files. The resulting output file included the function of each SNP, gene name, chromosome number, start position, end position, reference and alternative alleles, odds ratio, standard error, and *p* value for each variant.

Following functional annotation, a file containing the 43 gene names included in the NETRIN1 signaling pathway was used as an input to extract gene-based SNPs located in the pathway. For the genomic-PRSs, all gene-based SNPs, excluding those implicated in the NETRIN1 signaling pathway, were extracted. The two files were then used as input for the creation of PRSs.

### Genotyping and PRS Profiling

A total of 488,363 UKB blood samples (*n*_female_ = 264,857; *n*_male_ = 223,506; http://biobank.ctsu.ox.ac.uk/crystal/field.cgi?id=22001) were genotyped using two different arrays: UK BiLEVE array (*n* = 49,949) (http://biobank.ctsu.ox.ac.uk/crystal/refer.cgi?id=149600) and UKB Axiom array (*n* = 438,417) (http://biobank.ctsu.ox.ac.uk/crystal/refer.cgi?id=149601). Details of genotyping and quality control are described in more detail by Hagenaars *et al.* (25) and Bycroft *et al.* (26).

Using the largest available GWAS of MDD, PRSs for each individual were computed using PRSice (27) at five *p* value thresholds (0.01, 0.05, 0.1, 0.5, and 1) by adding the number of risk alleles and weighting them by the strength of association with MDD. PRSs were created both from SNPs annotated to the NETRIN1 signaling pathway and from SNPs from the rest of the genome, thus resulting in separate PRS lists. PRSs were created both with and without clump-based pruning of SNPs in linkage disequilibrium (*R*^2^ = .25, 250-kb window). The primary analysis reported in this manuscript concerns unpruned SNPs, owing to the potential of causal variants within the NETRIN1 pathway to be in linkage disequilibrium with other variants, and uses SNPs that met a significance level of *p* = .5, which is in line with previous studies 28, 29. Secondary analyses with other PRS *p* value thresholds, as well as with linkage disequilibrium pruned SNPs, are presented in Supplemental Tables S6–S21.

### Magnetic Resonance Imaging Acquisition

In this study, imaging-derived phenotypes produced by the UKB were used. Magnetic resonance imaging acquisition and preprocessing procedures for FA and MD values of white matter tracts were performed by the UKB using standardized protocols (https://biobank.ctsu.ox.ac.uk/crystal/docs/brain_mri.pdf). Images were collected on a single Siemens Skyra 3.0T scanner (Siemens, Erlangen, Germany) with a standard Siemens 32-channel head coil and were preprocessed using FSL packages; parcellation of white matter tracts was conducted using AutoPtx (30).

Summary data were composed of tract-averaged FA and MD values for 15 major white matter tracts, of which 12 are bilateral and three are unilateral. The white matter tracts were also categorized into three separate subsets: association fibers (inferior fronto-occipital fasciculus, uncinate fasciculus, cingulum bundle [gyrus and parahippocampal], and superior and inferior longitudinal fasciculus), thalamic radiation fibers (anterior, superior, and posterior thalamic radiations), and projection fibers (forceps major and minor, corticospinal tract, acoustic radiation, medial lemniscus, and middle cerebellar peduncle). Global measures of FA and MD are referred to as general factors of FA and MD.

Exclusion criteria comprised removal of scans with severe normalization problems by the UKB. Moreover, individuals whose FA and MD values were >3 SDs from the sample mean were also excluded. Results for the full data set with outliers included are also presented in Supplemental Tables S1 and S2. Lastly, because the position of the head and radio-frequency coil in the scanner may affect data quality as well as imaging-derived phenotypes, three scanner brain-position variables that may be used as confounding variables in subsequent analyses were generated by the UKB: lateral brain position X (http://biobank.ctsu.ox.ac.uk/crystal/field.cgi?id=25756), transverse brain position Y (http://biobank.ctsu.ox.ac.uk/crystal/field.cgi?id=25757), and longitudinal brain position Z (http://biobank.ctsu.ox.ac.uk/crystal/field.cgi?id=25758). The three variables were included as covariates in the statistical analysis described below.

### Statistical Methods

All analyses were conducted using R (version 3.2.3) in a Linux environment. To test the association between the NETRIN1 signaling pathway– and genomic pathway–derived unpruned PRS lists, we used repeated-measures linear mixed-effects models (function lme in package nlme) for 12 bilateral brain regions, correcting for hemisphere, with age, age^2^, gender, 15 genetic principal components, three magnetic resonance imaging head-position coordinates, and a genotype array set as covariates. For unilateral tracts, global measures of FA and MD, and tract categories, we used a general linear model (function lm), using the same covariates as above, and without hemisphere included as a separate term in the model. All models included both the genomic-PRS and the NETRIN1-PRS as predictor variables.

First, we tested the association between unpruned PRSs (both NETRIN1-PRSs and genomic-PRSs) and global white matter integrity. We applied principal component analysis on the 27 white matter tracts (12 tracts in both the right and left hemisphere and three unilateral tracts) to extract a latent measure. Scores of the first unrotated component of FA and MD (variance explained = 37.52% for FA and 38.83% for MD) were extracted and set as the dependent variable in a general linear model to test association with both NETRIN1-PRS and genomic-PRS.

We then examined the three categories of white matter tracts by applying principal component analysis on the regions involved in each, as a substantial proportion of white matter microstructural properties shows substantial commonality across these pathways (31). Scores of the first unrotated component of FA and MD were similarly extracted and set as dependent variables in general linear modeling, as above. Variance explained for each white matter tract subset was as follows: association fibers: 45.36% (FA), 50.76% (MD); thalamic radiations: 60.85% (FA), 73.40% (MD); projection fibers: 35.54% (FA), 29.28% (MD).

Lastly, we tested the association between PRSs (both NETRIN1-PRSs and genomic-PRSs) and each individual white matter tract (*N* = 15). We used a repeated-effect linear model for the 12 bilateral tracts and a random-effect general linear model for the three unilateral tracts.

False discovery rate correction was applied separately for the 15 individual tracts and for global and tract category values.

### Permutation Analysis

To establish that the effect of the NETRIN1 pathway–derived PRS on white matter integrity as measured by FA and MD was not due to chance, a circular genomic permutation was applied to the pathway SNP genotypes (32). This was done by placing all SNPs in the whole genome (excluding those in the NETRIN1 pathway) in a circular genome, according to their location. We then circularly permuted 1000 SNP lists with the same set size as the NETRIN1 pathway. We created 1000 PRS lists that were then fitted in linear mixed-effects and general linear models, depending on the white matter tract tested, and their association with five white matter tracts and one tract category, found to be significantly associated with NETRIN1, was tested.

## Results

**Figure 1:**
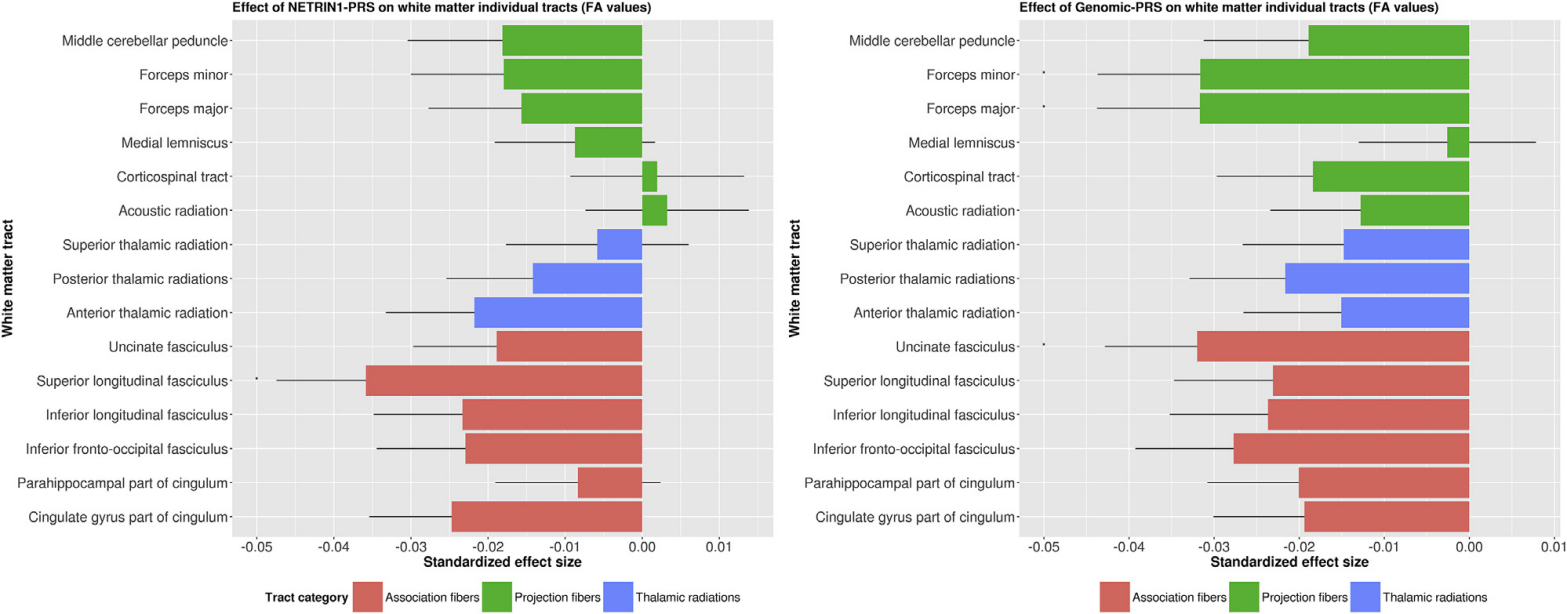
The effects of (left panel) major depressive disorder polygenic risk score (PRS) derived from the NETRIN1 signaling pathway (NETRIN1-PRS) and (right panel) major depressive disorder PRS derived from the whole genome, excluding NETRIN1 pathway genes (genomic-PRS), on fractional anisotropy (FA) values of white matter tracts. The x-axis indicates the standardized effect size of each pathway's PRSs, and the y-axis indicates the white matter tracts. The legend indicates the tract category belonging to each white matter tract. The error bar represents the SD of the mean.

**Figure 2:**
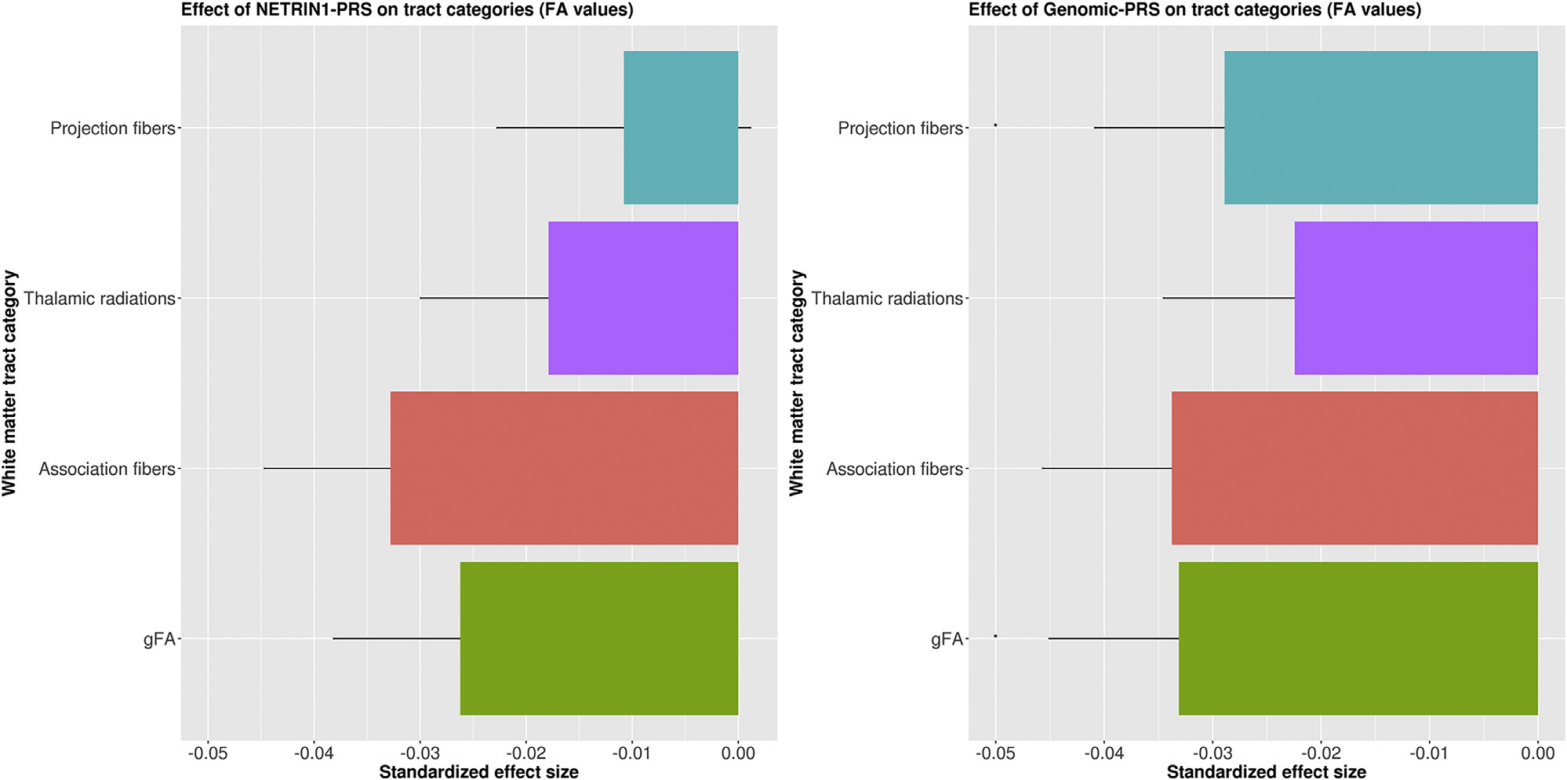
The effects of (left panel) major depressive disorder polygenic risk score (PRS) derived from the NETRIN1 signaling pathway (NETRIN1-PRS) and (right panel) major depressive disorder PRS derived from the whole genome, excluding NETRIN1 pathway genes (genomic-PRS), on fractional anisotropy (FA) values of tract categories and global FA (gFA). The x-axis indicates the standardized effect size of each pathway's PRS, and the y-axis indicates the tract categories. The error bar represents the SD of the mean.

**Figure 3:**
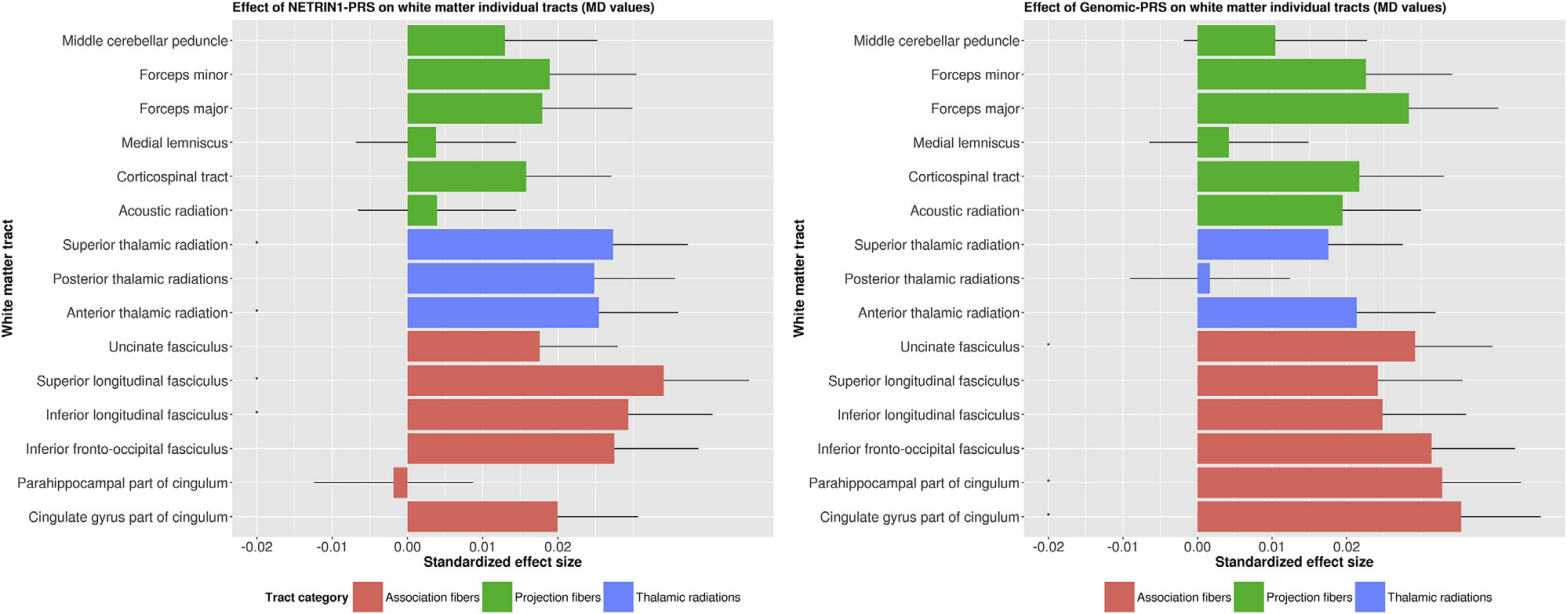
The effects of (left panel) major depressive disorder polygenic risk score (PRS) derived from the NETRIN1 signaling pathway (NETRIN1-PRS) and (right panel) major depressive disorder (PRS) derived from the whole genome, excluding NETRIN1 pathway genes (genomic-PRS), on mean diffusivity (MD) values of white matter tracts. The x-axis indicates the standardized effect size of each pathway's PRS, and the y-axis indicates the white matter tracts. The legend indicates the tract category belonging to each white matter tract. The error bar represents the SD of the mean.

**Figure 4:**
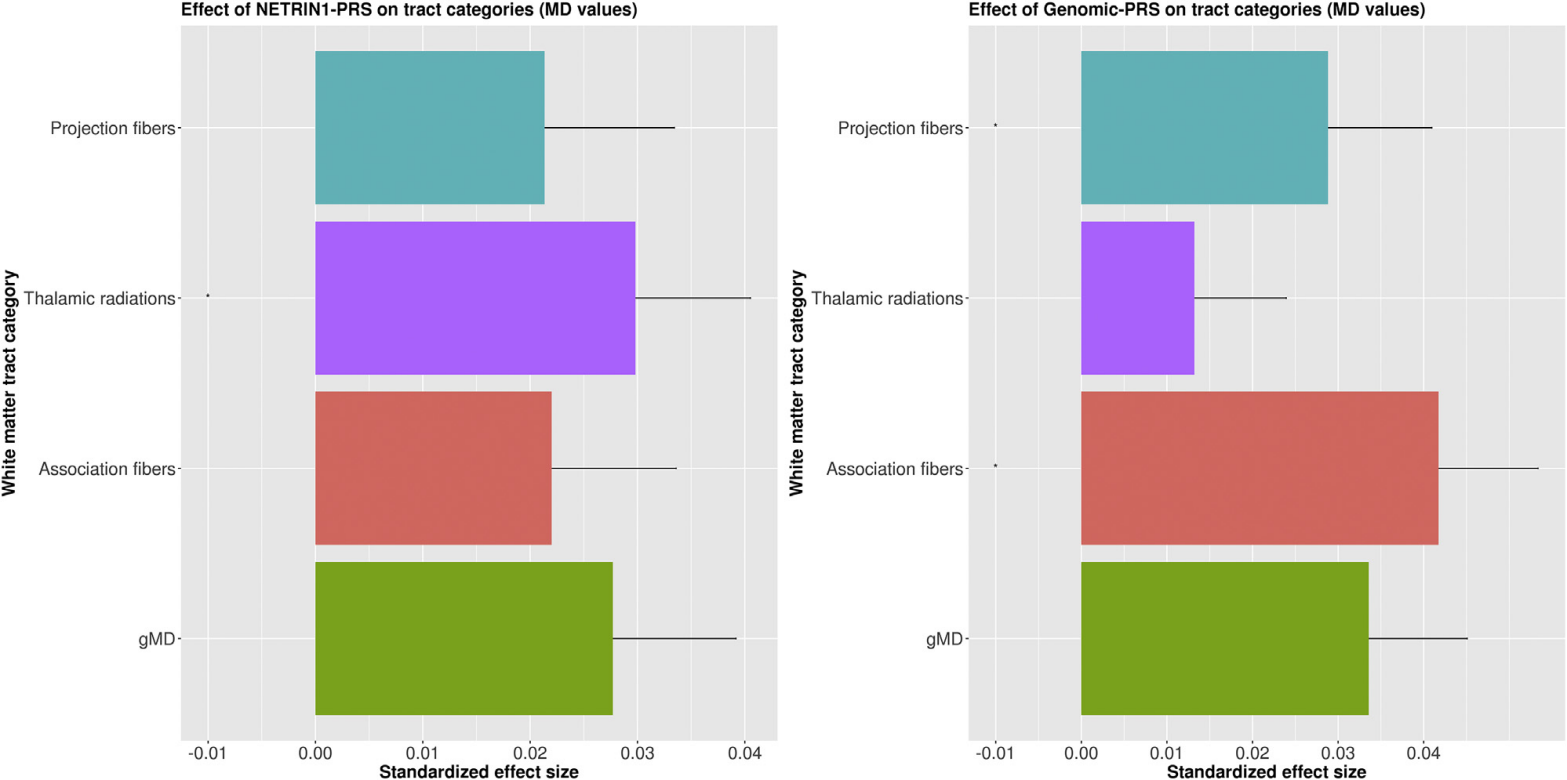
The effects of (left panel) major depressive disorder PRS derived from the NETRIN1 signaling pathway (NETRIN1-PRS) and (right panel) major depressive disorder PRS derived from the whole genome, excluding NETRIN1 pathway genes (genomic-PRS), on mean diffusivity (MD) values of tract categories and global MD (gMD). The x-axis indicates the standardized effect size of each pathway's PRS, and the y-axis indicates the tract categories. The error bar represents the SD of the mean.

Results presented below are significant specifically to each pathway. White matter tracts showing a significant association with both the NETRIN1-PRS and the genomic-PRS pathways are described in the Supplement. Results for all individual white matter tracts, tract categories, and global measures can be found in Table 1, Table 2, Table 3, Table 4 and Figure 1, Figure 2, Figure 3, Figure 4.

**Table 1:** The Effect of Major Depressive Disorder NETRIN1-PRS and Genomic-PRS at PRS Threshold 0.5 on Individual White Matter Tracts (Fractional Anisotropy Values)

White Matter Tracts	NETRIN1-PRS						Genomic-PRS					
	Effect Size, β	SD	*t* Value	*p* Value	*p* Value, FDR Corrected	*R*^2^^a^	Effect Size, β	SD	*t* Value	*p* Value	*p* Value, FDR Corrected	*R*^2^^a^
Association Fibers												
Cingulate gyrus part of cingulum	−.025	.011	−2.323	.020	.152	.062	−.019	.011	−1.817	.069	.115	.038
Parahippocampal part of cingulum	−.008	.011	−0.780	.435	.544	.007	−.020	.011	−1.873	.061	.115	.040
Inferior fronto-occipital fasciculus	−.023	.011	−1.997	.046	.172	.053	−.028	.012	−2.409	.016	.060	.076
Inferior longitudinal fasciculus	−.023	.011	−2.029	.043	.172	.054	−.024	.012	−2.056	.040	.115	.056
Superior longitudinal fasciculus	−.036	.012	−3.093	.002	.030^b^	.128	−.023	.012	−1.988	.047	.115	.053
Uncinate fasciculus	−.019	.011	−1.747	.081	.202	.102	−.032	.011	−2.954	.003	.043^b^	.102
Thalamic Radiations												
Anterior thalamic radiation	−.022	.011	−1.900	.057	.172	.048	−.015	.011	−1.310	.190	.238	.023
Posterior thalamic radiations	−.014	.011	−1.267	.205	.308	.020	−.022	.011	−1.929	.054	.115	.047
Superior thalamic radiation	−.006	.012	−0.493	.622	.718	.003	−.015	.012	−1.246	.213	.244	.022
Projection Fibers												
Acoustic radiation	.003	.011	0.306	.759	.814	.001	−.013	.011	−1.207	.228	.244	.016
Corticospinal tract	.002	.011	0.173	.863	.863	.000	−.018	.011	−1.632	.103	.154	.034
Medial lemniscus	−.009	.010	−0.842	.400	.544	.008	−.003	.010	−0.249	.803	.803	.001
Forceps major	−.016	.012	−1.301	.193	.308	.024	−.032	.012	−2.626	.009	.043^b^	.100
Forceps minor	−.018	.012	−1.496	.135	.262	.032	−.032	.012	−2.628	.009	.043^b^	.099
Middle cerebellar peduncle	−.018	.012	−1.476	.140	.262	.032	−.019	.012	−1.536	.125	.170	.035

FDR, false discovery rate; Genomic-PRS, polygenic risk score derived from the whole genome, excluding NETRIN1 pathway genes; NETRIN1-PRS, polygenic risk score derived from the NETRIN1 signaling pathway; PRS, polygenic risk score.

a *R*^2^ is the estimate of variance explained by each pathway, in %.

b Statistically significant *p* value after false discovery rate correction.

**Table 2:** The Effect of Major Depressive Disorder Polygenic Risk Scores Derived From the NETRIN1 Signaling Pathway and the Whole Genome, Excluding NETRIN1 Pathway Genes, at Polygenic Risk Score Threshold 0.5 on Global Fractional Anisotropy and Three White Matter Tract Categories

	NETRIN1-PRS						Genomic-PRS					
	Effect Size, β	SD	*t* Value	*p* Value	*p* Value, FDR Corrected	*R*^2^^a^	Effect Size, β	SD	*t* Value	*p* Value	*p* Value, FDR Corrected	*R*^2^^a^
gFA	−.026	.012	−2.197	.028	.056	.068	−.033	.012	-2.769	.006	.011^b^	.109
Association Fibers	−.033	.012	−2.762	.006	.023	.107	−.034	.012	-2.836	.005	.011	.113
Thalamic Radiations	−.018	.012	−1.482	.138	.185	.032	−.022	.012	-1.855	.064	.064	.050
Projection Fibers	−.011	.012	−0.904	.366	.366	.012	−.029	.012	-2.415	.016	.021^b^	.083

FDR, false discovery rate; Genomic-PRS, polygenic risk score derived from the whole genome, excluding NETRIN1 pathway genes; gFA, global fractional anisotropy; NETRIN1-PRS, polygenic risk score derived from the NETRIN1 signaling pathway; PRS, polygenic risk score.

a *R*^2^ is the estimate of variance explained by each pathway, in %.

b Statistically significant *p* value after false discovery rate correction.

**Table 3:** The Effect of NETRIN1-PRS and Genomic-PRS at PRS Threshold 0.5 on Individual White Matter Tracts (Mean Diffusivity Values)

White Matter Tracts	NETRIN1-PRS						Genomic-PRS					
	Effect Size, β	SD	*t* Value	*p* Value	*p* Value, FDR Corrected	*R*^2^^a^	Effect Size, β	SD	*t* Value	*p* Value	*p* Value, FDR Corrected	*R*^2^^a^
Association Fibers												
Cingulate gyrus part of cingulum	.020	.011	1.877	.061	.130	.040	.035	.011	3.317	.001	.014^b^	.124
Parahippocampal part of cingulum	−.002	.011	−0.175	.861	.861	.000	.033	.011	3.109	.002	.014^b^	.107
Inferior fronto-occipital fasciculus	.027	.011	2.461	.014	.047	.075	.031	.011	2.807	.005	.019	.098
Inferior longitudinal fasciculus	.029	.011	2.625	.009	.043^b^	.086	.025	.011	2.216	.027	.067	.061
Superior longitudinal fasciculus	.034	.011	3.009	.003	.039^b^	.116	.024	.011	2.133	.033	.071	.058
Uncinate fasciculus	.018	.010	1.698	.090	.168	.085	.029	.010	2.815	.005	.019^b^	.084
Thalamic Radiations												
Anterior thalamic radiation	.025	.011	2.420	.016	.047^b^	.065	.021	.011	2.028	.043	.080	.046
Posterior thalamic radiations	.025	.011	2.326	.020	.050	.062	.002	.011	0.157	.876	.876	.000
Superior thalamic radiation	.027	.010	2.758	.006	.043^b^	.074	.018	.010	1.771	.077	.096	.031
Projection Fibers												
Acoustic radiation	.004	.010	0.375	.708	.772	.002	.019	.011	1.853	.064	.087	.038
Corticospinal tract	.016	.011	1.399	.162	.221	.025	.022	.011	1.921	.055	.082	.047
Medial lemniscus	.004	.011	0.357	.721	.772	.001	.004	.011	0.396	.692	.741	.002
Forceps major	.018	.012	1.495	.135	.203	−.026	.028	.012	2.362	.018	.055	.019
Forceps minor	.019	.012	1.640	.101	.168	−.063	.023	.012	1.958	.050	.082	−.051
Middle cerebellar peduncle	.013	.012	1.058	.290	.363	.016	.010	.012	0.852	.394	.455	.010

FDR, false discovery rate; PRS, polygenic risk score.

a *R*^2^ is the estimate of variance explained by each pathway, in %.

b Statistically significant *p* value after false discovery rate correction.

**Table 4:** The Effect of NETRIN1-PRS and Genomic-PRS at PRS Threshold 0.5 on Global Mean Diffusivity and Three White Matter Tract Subsets

	NETRIN1-PRS						Genomic-PRS					
	Effect Size, β	SD	*t* Value	*p* Value	*p* Value, FDR Corrected	*R*^2^^a^	Effect Size, β	SD	*t* Value	*p* Value	*p* Value, FDR Corrected	*R*^2^^a^
gMD	.028	0.011	2.417	.016	.031	.076	.034	.011	2.924	.003	.007	.111
Association Fibers	.022	0.012	1.897	.058	.077	.048	.042	.012	3.591	.000	.001^b^	.172
Thalamic Radiations	.030	0.011	2.785	.005	.021^b^	.089	.013	.011	1.232	.218	.218	.017
Projection Fibers	.021	0.012	1.766	.077	.077	.045	.029	.012	2.380	.017	.023^b^	.081

FDR, false discovery rate; Genomic-PRS, polygenic risk score derived from the whole genome, excluding NETRIN1 pathway genes; gMD, global mean diffusivity; NETRIN1-PRS, polygenic risk score derived from the NETRIN1 signaling pathway; PRS, polygenic risk score.

a *R*^2^ is the estimate of variance explained by each pathway, in %.

b Statistically significant *p* values after false discovery rate correction.

### The Effect of Unpruned NETRIN1-PRS and Genomic-PRS on FA Measures of White Matter Integrity

#### Global Measures

We first tested the effect of NETRIN1-PRS and genomic-PRS on global FA. Lower global FA was significantly associated with higher genomic-PRS (β = −.033, *p*_corrected_ = .011) only.

#### Tract Categories

We then tested the association between NETRIN1-PRS and genomic-PRS and three subsets of white matter tracts (association fibers, thalamic radiations, and projection fibers). Significantly lower FA values in projection fibers were found for genomic-PRS (β = −.028, *p*_corrected_ = .020) only.

#### Individual White Matter Tracts

Lastly, we investigated the effect of NETRIN1-PRS and genomic-PRS on white matter integrity in 15 individual white matter tracts. NETRIN1-PRS, but not genomic-PRS, was associated with significantly lower FA in the superior longitudinal fasciculus (β = −.035, *p*_corrected_ = .029).

In the genomic-PRS, we found significantly lower FA in the forceps major (β = −.031, *p*_corrected_ = .043), forceps minor (β = −.031, *p*_corrected_ = .043) and uncinate fasciculus (β = −.031, p_corrected_ = .043). None of these tracts showed significant associations with NETRIN1-PRS.

### The Effect of Unpruned NETRIN1-PRS and Genomic-PRS on MD Measures of White Matter Integrity

#### Tract Categories

MD values for association fibers (β = .041, *p*_corrected_ = .001) and projection fibers (β = .028, *p*_corrected_ = .023) were found to be significantly higher for genomic-PRS, but not NETRIN1-PRS. MD values for thalamic radiations were found to be significantly higher in the NETRIN1-PRS (β = .029, *p*_corrected_ = .021), whereas there was no significant association with genomic-PRS.

#### Individual White Matter Tracts

Within the 15 individual white matter tracts, we found numerous areas significantly associated with both the NETRIN1-PRS and genomic-PRS. With regards to NETRIN1-PRS, MD values were significantly higher in the inferior longitudinal fasciculus (β = .029, *p*_corrected_ = .043), superior longitudinal fasciculus (β = .034, *p*_corrected_ = .039), and anterior (β = .025, *p*_corrected_ = .046) and superior (β = .027, *p*_corrected_ = .043) thalamic radiations. All of these significant associations were specific for NETRIN1-PRS.

In the genomic-PRS, we found significantly higher MD values in the cingulate gyrus (β = .035, *p*_corrected_ = .013) and parahippocampal part of cingulum (β = .032, *p*_corrected_ = .014) and in the uncinate fasciculus (β = .029, *p*_corrected_ = .018).

### Permutation Analysis

NETRIN1-PRSs, but not genomic-PRSs, were found to be individually significantly associated with white matter microstructure in the following white matter tracts: superior longitudinal fasciculus, as measured by lower FA, and superior and inferior longitudinal fasciculus and anterior and superior thalamic radiations, as well as thalamic radiations tract category, as measured by higher MD. Therefore, we additionally performed a circular genomic permutation analysis and found that the variance explained by NETRIN1-PRSs in these tracts was significantly higher than that expected by chance (Table 5).

**Table 5:** Permutation Results for NETRIN1-PRS at PRS Threshold 0.5 on Five Significant White Matter Tracts and One Significant Tract Category

White Matter Tract or Tract Category	Effect Size of Regression NETRIN1 Pathway	Regression NETRIN1 Pathway *t* Score	NETRIN1 Calculated Permutation *p* Value
Superior Longitudinal Fasciculus (FA)	−.035	−3.093	.004
Superior Longitudinal Fasciculus (MD)	.034	3.008	.004
Inferior Longitudinal Fasciculus (MD)	.029	2.624	.014
Anterior Thalamic Radiations (MD)	.025	2.419	.023
Superior Thalamic Radiations (MD)	.027	2.757	.007
Thalamic Radiations (MD)	.029	2.785	.008

FA, fractional anisotropy; MD, mean diffusivity; NETRIN1-PRS, polygenic risk score derived from the NETRIN1 signaling pathway; PRS, polygenic risk score.

## Discussion

In this study, we aimed to investigate whether PRSs calculated from the NETRIN1 signaling pathway are significantly and specifically associated with white matter integrity while simultaneously modeling genomic-PRS in >6000 individuals. We found significant differences in white matter integrity in both NETRIN1-PRSs and genomic-PRSs, for both FA and MD values. Regarding FA values, for NETRIN1-PRSs but not for genomic-PRSs, a significant association was observed in the superior longitudinal fasciculus. NETRIN1-PRSs alone were significantly associated with higher generalized thalamic radiations as measured by MD, as well as higher MD in the superior and inferior longitudinal fasciculus and the anterior and superior thalamic radiations. Genomic-PRSs were also significantly associated with FA and MD values in several tracts.

One of the main findings in our paper was both a reduction of FA and an increase of MD in the superior longitudinal fasciculus in relation to NETRIN1-PRS. The superior longitudinal fasciculus, a tract in association fibers, connects the frontal, temporal, parietal, and occipital lobes and has been shown to be highly involved in MDD 33, 34. FA reductions in the superior longitudinal fasciculus have also been found in previous studies combining genetic and neuroimaging techniques (35), further indicating that the tract might be an important biomarker of MDD. In addition to this finding, we also found an increase in MD values in the inferior longitudinal fasciculus, a tract connecting the temporal and occipital lobes. Key areas in these two lobes include the amygdala and hippocampus, which are known to be implicated in emotion processing, a process that is disrupted in MDD (36). Previous studies have found disrupted white matter integrity in this tract in association with MDD using FA, indicating that it may play an important role in the pathophysiology of MDD (35).

We also found an MD increase in the thalamic radiations tract category. Thalamic radiations connect the thalamus with numerous cortical areas (37) and are connected to various cognitive processes, such as attention and wakefulness (38). Thalamocortical axons play an important role during development, as their projection from the dorsal thalamus (DT) transmits sensory information to the neocortex (37). Thalamic radiations have previously been linked to MDD in numerous studies. For instance, a decrease in FA was found in the thalamic radiations subset in a large UKB sample comparing 335 MDD patients with 754 healthy individuals (20). This tract subset was also found to be significantly associated with higher PRS, indicating that there is a link between the sets of tracts and a potential genetic predisposition to MDD (35).

NETRIN1 and its receptor DCC, one of the proteins in the NETRIN1-pathway, have been previously implicated in thalamic axonal growth. NETRIN1 promotes growth of thalamocortical axons by binding to and activating DCC, which is expressed in the DT. Moreover, NETRIN1 has been shown to enhance axonal growth in explants of the DT, as well as to provide guidance from the DT to the cortex (37). It has also been found that serotonin, which is highly implicated in MDD, modulates the effect of NETRIN1 on embryonic thalamocortical axons 37, 38, 39. The active involvement of NETRIN1 in thalamocortical axonal growth, therefore, may explain our findings, and it further confirms that there is a potential link between a biological pathway and specific neurobiological markers in MDD.

Several other tracts also showed a significant association of FA (individually in forceps major and minor and uncinate fasciculus, and in global measures of FA and projection fibers) and MD (individually in the cingulate part of the cingulum, parahippocampal part of cingulum, and uncinate fasciculus and in global measures of association and projection fibers) with genomic-PRS, most of which have also been previously associated with MDD 20, 35. This evidence further confirms that there is an association between genetic predisposition to MDD and disruptions in white matter integrity, also for variants that lie outside the NETRIN1-DCC pathway. As such, these findings suggest that both PRS lists affect integrity across the white matter tracts, each with localized, pronounced effects in specific tracts.

The current study has several strengths and a few potential limitations. First, to our knowledge it is the largest combined genetic and neuroimaging study investigating the effect of PRS derived from a specific biological pathway on white matter integrity. Moreover, our analysis consisted of a population-based sample of ambulant individuals recruited to the UKB. Our findings might therefore be robust and generalizable to other samples within a certain age range, although studies such as the UKB are not immune to biases associated with study participation, such as collider bias (40).

In addition to the large sample, the fact that NETRIN1-PRS are derived from only 43 genes, comprising approximately 0.215% of the genes in the whole genome (*N* ≈ 20,000), suggests that MDD risk–associated variation exerts a disproportionate influence on white matter microstructure. Our findings are further supported by permutation analysis. The association between the NETRIN1 pathway and white matter integrity is therefore likely to reflect the importance of a specific pathway in the pathophysiology of MDD.

The NETRIN1 signaling pathway has previously been found to be implicated in MDD (14). In the current study, we were able to find specific neurobiological structural connectivity markers associated with this biological pathway. To our knowledge, the current study is the first one to note an association between PRSs derived specifically from the NETRIN1 signaling pathway and several white matter tracts in a large genetic and neuroimaging data set. This indicates that these brain structures may be involved in the manifestation of genetic risk of MDD and ultimately the etiology of the disorder.
