# Supplementary material for: Association of Whole-Genome and NETRIN1 Signaling Pathway–Derived Polygenic Risk Scores for Major Depressive Disorder and White Matter Microstructure in the UK Biobank
Source: Biol Psychiatry Cogn Neurosci Neuroimaging. 2019 Jan;4(1):91–100. doi: 10.1016/j.bpsc.2018.07.006 (PMC6374980; doi:10.1016/j.bpsc.2018.07.006)
Supplement: Supplemental Material [file mmc1.pdf]

Association of Whole-genome and NETRIN1 Signaling Pathway-derived Polygenic Risk Scores for Major Depressive Disorder and White Matter Microstructure in UK Biobank

Supplemental Information

Supplementary notes

- Demographic data concerning complete dataset of individuals with DTI values
- Descriptive statistics of imaging phenotype
- NETRIN1 signalling pathway gene list
- Demographic data and FA descriptive statistics of individuals excluded from the study (N = 19)
- Demographic data and MD descriptive statistics of individuals excluded from the study (N = 30)
- Statistical analysis of FA and MD values containing:
    1. Unpruned NETRIN1- and genomic-PRS with outliers excluded (6,401 for FA and 6,390 for MD) at all 5 thresholds (0.01, 0.05, 0.1, 0.5, 1) and full sample (6,420) at threshold 0.5
    2. Pruned NETRIN1- and Genomic-PRS with outliers excluded (6,401 for FA and 6,390 for MD) at all 5 thresholds (0.01, 0.05, 0.1, 0.5, 1) and full sample (6,420) at threshold 0.5
- White matter tracts significantly associated with both NETRIN1-PRS and genomic-PRS.
    1. Fractional anisotropy
    2. Mean diffusivity
- Data analysis code – example

Supplementary tables

- Table S1. Descriptive statistics of FA values (mean and standard deviation). The full dataset contains 6,420 individuals, while the outliers-excluded dataset contains 6,401 individuals.

- Table S2. Descriptive statistics of MD values (mean and standard deviation). The full dataset contains 6,420 individuals, while the outliers-excluded dataset contains 6,390 individuals.

- Table S3. Gene list and brief gene description included in the NETRIN1 signalling pathway, composed of 43 genes.

- Table S4. Descriptive statistics of FA values (mean and standard deviation) for individuals excluded from the study (N = 19).

- Table S5. Descriptive statistics of MD values (mean and standard deviation) for individuals excluded from the study (N = 30).

- Table S6. The effect of unpruned NETRIN1- and Genomic-PRS at thresholds 0.01, 0.05, 0.1, 0.5 and 1 on individual white matter tracts (FA) (N = 6,401).

- Table S7. The effect of unpruned NETRIN1- and Genomic-PRS at thresholds 0.01, 0.05, 0.1, 0.5 and 1 on tract categories (FA) (N = 6,401).

- Table S8. The effect of unpruned NETRIN1- and Genomic-PRS at threshold 0.5 on individual white matter tracts (FA) (N = 6,420).

- Table S9. The effect of unpruned NETRIN1- and Genomic-PRS at threshold 0.5 on tract categories (FA) (N = 6,420).

- Table S10. The effect of unpruned NETRIN1- and Genomic-PRS at thresholds 0.01, 0.05, 0.1, 0.5 and 1 on individual white matter tracts (MD) (N = 6,390).

- Table S11. The effect of unpruned NETRIN1- and Genomic-PRS at thresholds 0.01, 0.05, 0.1, 0.5 and 1 on tract categories (MD) (N = 6,390).

- Table S12. The effect of unpruned NETRIN1- and Genomic-PRS at threshold 0.5 on individual white matter tracts (MD) (N = 6,420).

- Table S13. The effect of unpruned NETRIN1- and Genomic-PRS at threshold 0.5 on tract categories (MD) (N = 6,420).

- Table S14. The effect of pruned NETRIN1- and Genomic-PRS at thresholds 0.01, 0.05,

0.1, 0.5 and 1 on individual white matter tracts (FA) (N = 6,401).

- Table S15. The effect of pruned NETRIN1- and Genomic-PRS at thresholds 0.01, 0.05, 0.1, 0.5 and 1 on tract categories (FA) (N = 6,401).
- Table S16. The effect of pruned NETRIN1- and Genomic-PRS at threshold 0.5 on individual white matter tracts (FA) (N = 6,420).
- Table S17. The effect of pruned NETRIN1- and Genomic-PRS at threshold 0.5 on tract categories (FA) (N = 6,420).
- Table S18. The effect of pruned NETRIN1- and Genomic-PRS at thresholds 0.01, 0.05, 0.1, 0.5 and 1 on individual white matter tracts (MD) (N = 6,390).
- Table S19. The effect of pruned NETRIN1- and Genomic-PRS at thresholds 0.01, 0.05, 0.1, 0.5 and 1 on tract categories (MD) (N = 6,390).
- Table S20. The effect of pruned NETRIN1- and Genomic-PRS at threshold 0.5 on individual white matter tracts (MD) (N = 6,420).
- Table S21. The effect of pruned NETRIN1- and Genomic-PRS at threshold 0.5 on tract categories (MD) (N = 6,420).

Table S1. Descriptive statistics of FA values (mean and standard deviation). The full dataset contains 6,420 individuals, while the outliers-excluded dataset contains 6,401 individuals.

Demographic data concerning complete dataset of individuals with DTI values

Complete dataset (N = 6,420): N female = 3,345; N male = 3,075; mean age: 62.62 +/- 7.37 years; age range: 45.92 – 78.42

Descriptive statistics of imaging phenotype

Fractional Anisotropy				
	Full dataset (N = 6,420)		Outliers excluded dataset (N = 6,401)	
White matter tract	Mean	SD	Mean	SD
Cingulate gyrus part of cingulum (left)	0.535	0.035	0.535	0.033
Cingulate gyrus part of cingulum (right)	0.497	0.034	0.498	0.033
Parahippocampal part of cingulum (left)	0.314	0.029	0.314	0.028
Parahippocampal part of cingulum (right)	0.313	0.030	0.313	0.030
Inferior fronto-occipital fasciculus (left)	0.475	0.024	0.476	0.022
Inferior fronto-occipital fasciculus (right)	0.465	0.021	0.465	0.020
Inferior longitudinal fasciculus (left)	0.460	0.021	0.460	0.019
Inferior longitudinal fasciculus (right)	0.451	0.020	0.451	0.018
Superior longitudinal fasciculus (left)	0.440	0.022	0.440	0.020
Superior longitudinal fasciculus (right)	0.423	0.021	0.424	0.019
Uncinate fasciculus (left)	0.388	0.024	0.388	0.235
Uncinate fasciculus (right)	0.390	0.021	0.390	0.020
Anterior thalamic radiation (left)	0.399	0.019	0.399	0.017
Anterior thalamic radiation (right)	0.392	0.019	0.392	0.017
Posterior thalamic radiation (left)	0.458	0.022	0.458	0.020
Posterior thalamic radiation (right)	0.455	0.022	0.456	0.020
Superior thalamic radiation (left)	0.422	0.019	0.423	0.018
Superior thalamic radiation (right)	0.422	0.020	0.422	0.018
Acoustic radiation (left)	0.419	0.023	0.420	0.021
Acoustic radiation (right)	0.411	0.022	0.412	0.020
Corticospinal tract (left)	0.545	0.024	0.545	0.022
Corticospinal tract (right)	0.539	0.025	0.539	0.022
Medial lemniscus (left)	0.419	0.024	0.419	0.023
Medial lemniscus (right)	0.422	0.025	0.422	0.024
Forceps major	0.580	0.029	0.580	0.027
Forceps minor	0.465	0.022	0.465	0.020
Middle cerebellar peduncle	0.481	0.031	0.481	0.029

Table S2. Descriptive statistics of MD values (mean and standard deviation). The full dataset contains 6,420 individuals, while the outliers-excluded dataset contains 6,390 individuals.

Mean Diffusivity				
	Full dataset (N = 6,420)		Outliers excluded dataset (N = 6,390)	
White matter tract	Mean	SD	Mean	SD
Cingulate gyrus part of cingulum (left)	0.0007	0.00003	0.0007	0.00002
Cingulate gyrus part of cingulum (right)	0.0007	0.00003	0.0007	0.00002
Parahippocampal part of cingulum (left)	0.0008	0.00006	0.0008	0.00005
Parahippocampal part of cingulum (right)	0.0008	0.00006	0.0008	0.00005
Inferior fronto-occipital fasciculus (left)	0.0008	0.00003	0.0008	0.00003
Inferior fronto-occipital fasciculus (right)	0.0008	0.00003	0.0008	0.00003
Inferior longitudinal fasciculus (left)	0.0008	0.00003	0.0008	0.00003
Inferior longitudinal fasciculus (right)	0.0008	0.00003	0.0008	0.00003
Superior longitudinal fasciculus (left)	0.0007	0.00003	0.0007	0.00003
Superior longitudinal fasciculus (right)	0.0007	0.00003	0.0007	0.00003
Uncinate fasciculus (left)	0.0008	0.00004	0.0008	0.00003
Uncinate fasciculus (right)	0.0008	0.00003	0.0008	0.00003
Anterior thalamic radiation (left)	0.0007	0.00003	0.0007	0.00003
Anterior thalamic radiation (right)	0.0007	0.00003	0.0007	0.00003
Posterior thalamic radiation (left)	0.0008	0.00004	0.0008	0.00004
Posterior thalamic radiation (right)	0.0008	0.00004	0.0008	0.00004
Superior thalamic radiation (left)	0.0007	0.00003	0.0007	0.00002
Superior thalamic radiation (right)	0.0007	0.00003	0.0007	0.00002
Acoustic radiation (left)	0.0007	0.00004	0.0007	0.00003
Acoustic radiation (right)	0.0007	0.00004	0.0007	0.00003
Corticospinal tract (left)	0.0007	0.00002	0.0007	0.00002
Corticospinal tract (right)	0.0007	0.00002	0.0007	0.00002
Medial lemniscus (left)	0.0009	0.00004	0.0009	0.00003
Medial lemniscus (right)	0.0009	0.00004	0.0009	0.00003
Forceps major	0.0009	0.00005	0.0009	0.00005
Forceps minor	0.0008	0.00003	0.0008	0.00003
Middle cerebellar peduncle	0.0007	0.00006	0.0007	0.00006

Table S3. Gene list and brief gene description included in the NETRIN1 signalling pathway, composed of 43 genes.

NETRIN1 signalling pathway gene list

Gene name	Description
UNC5D	unc-5 homolog D (C. elegans)
HFE2	hemochromatosis type 2 (juvenile)
DCC	deleted in colorectal carcinoma
DOCK1	dedicator of cytokinesis 1
UNC5B	unc-5 homolog B (C. elegans)
ABLIM3	actin binding LIM protein family, member 3
FYN	FYN oncogene related to SRC, FGR, YES
RGMB	RGM domain family, member B
ABLIM1	actin binding LIM protein 1
MYO10	myosin X
NCK1	NCK adaptor protein 1
NEO1	neogenin 1
PITPNA	phosphatidylinositol transfer protein, alpha
PLCG1	phospholipase C, gamma 1
PRKCQ	protein kinase C, theta
RGMA	RGM domain family, member A
TRPC7	transient receptor potential cation channel
PTK2	PTK2 protein tyrosine kinase 2
RAC1	ras-related C3 botulinum toxin substrate 1 percursor
NTN4	netrin 4
ROBO1	roundabout, axon guidance receptor, homolog 1
SIAH1	seven in absentia homolog 1 (Drosophila)
SIAH2	seven in absentia homolog 2 (Drosophila)
SLIT1	slit homolog 1 (Drosophila)
SLIT3	slit homolog 3 (Drosophila)
SRC	v-src sarcoma (Schmidt-Ruppin A-2) viral oncogene
TRIO	triple functional domain (PTPRF interacting)
TRPC3	transient receptor potential cation channel
TRPC4	transient receptor potential cation channel
TRPC5	transient receptor potential cation channel
TRPC6	transient receptor potential cation channel
LOC730030	---
LOC730221	---
LOC730335	---
LOC730221	---
LOC730030	---
EZR	ezrin
UNC5C	unc-5 homolog C (C. elegans)
WASL	Wiskott-Aldrich syndrome-like
UNC5A	unc-5 homolog A (C. elegans)
SLIT2	slit homolog 2 (Drosophila)
NTN1	netrin 1
CDC42	cell division cycle 42 (GTP binding protein)

Table S4. Descriptive statistics of FA values (mean and standard deviation) for individuals excluded from the study (N = 19).

Demographic data and FA descriptive statistics of individuals excluded from the study (N = 19)

N female = 11; N male = 8; mean age: 69.26 +/- 4.53 years; age range: 58.92 – 77.42

Fractional Anisotropy		
	Outlier dataset (N = 19)	
White matter tract	Mean	SD
Cingulate gyrus part of cingulum (left)	0.407	0.149
Cingulate gyrus part of cingulum (right)	0.388	0.144
Parahippocampal part of cingulum (left)	0.246	0.091
Parahippocampal part of cingulum (right)	0.254	0.095
Inferior fronto-occipital fasciculus (left)	0.354	0.127
Inferior fronto-occipital fasciculus (right)	0.354	0.127
Inferior longitudinal fasciculus (left)	0.348	0.124
Inferior longitudinal fasciculus (right)	0.338	0.122
Superior longitudinal fasciculus (left)	0.325	0.117
Superior longitudinal fasciculus (right)	0.309	0.112
Uncinate fasciculus (left)	0.296	0.106
Uncinate fasciculus (right)	0.301	0.107
Anterior thalamic radiation (left)	0.306	0.110
Anterior thalamic radiation (right)	0.306	0.109
Posterior thalamic radiation (left)	0.358	0.127
Posterior thalamic radiation (right)	0.350	0.126
Superior thalamic radiation (left)	0.335	0.119
Superior thalamic radiation (right)	0.336	0.120
Acoustic radiation (left)	0.324	0.116
Acoustic radiation (right)	0.320	0.116
Corticospinal tract (left)	0.436	0.156
Corticospinal tract (right)	0.431	0.155
Medial lemniscus (left)	0.353	0.127
Medial lemniscus (right)	0.353	0.130
Forceps major	0.460	0.166
Forceps minor	0.346	0.125
Middle cerebellar peduncle	0.381	0.171

Table S5. Descriptive statistics of MD values (mean and standard deviation) for individuals excluded from the study (N = 30).

Demographic data and MD descriptive statistics of individuals excluded from the study (N = 30)

N female = 18; N male = 12; mean age: 70.29 +/- 4.66 years; age range: 58.92 – 77.42

Mean Diffusivity		
	Outlier dataset (N = 30)	
White matter tract	Mean	SD
Cingulate gyrus part of cingulum (left)	0.0007	0.0002
Cingulate gyrus part of cingulum (right)	0.0007	0.0002
Parahippocampal part of cingulum (left)	0.0009	0.0002
Parahippocampal part of cingulum (right)	0.0009	0.0002
Inferior fronto-occipital fasciculus (left)	0.0008	0.0002
Inferior fronto-occipital fasciculus (right)	0.0008	0.0002
Inferior longitudinal fasciculus (left)	0.0008	0.0002
Inferior longitudinal fasciculus (right)	0.0008	0.0002
Superior longitudinal fasciculus (left)	0.0008	0.0002
Superior longitudinal fasciculus (right)	0.0008	0.0002
Uncinate fasciculus (left)	0.0008	0.0002
Uncinate fasciculus (right)	0.0008	0.0002
Anterior thalamic radiation (left)	0.0008	0.0002
Anterior thalamic radiation (right)	0.0008	0.0002
Posterior thalamic radiation (left)	0.0009	0.0002
Posterior thalamic radiation (right)	0.0009	0.0002
Superior thalamic radiation (left)	0.0007	0.0002
Superior thalamic radiation (right)	0.0007	0.0002
Acoustic radiation (left)	0.0008	0.0002
Acoustic radiation (right)	0.0008	0.0002
Corticospinal tract (left)	0.0007	0.0002
Corticospinal tract (right)	0.0007	0.0002
Medial lemniscus (left)	0.0008	0.0002
Medial lemniscus (right)	0.0008	0.0002
Forceps major	0.0009	0.0002
Forceps minor	0.0008	0.0002
Middle cerebellar peduncle	0.0007	0.0002

Table S6. The effect of unpruned NETRIN1- and Genomic-PRS at thresholds 0.01, 0.05, 0.1, 0.5 and 1 on individual white matter tracts (FA) (N = 6,401).

Statistical analysis of FA and MD values containing:

Unpruned NETRIN1- and genomic-PRS with outliers included (6,420) and outliers excluded

(6,401 for FA and 6,390 for MD) at all 5 thresholds (0.01, 0.05, 0.1, 0.5, 1)

	Value	Std. Error	t-value	p-value
PGRS THRESHOLD: 0.01				
NETRIN1 acoustic_radiation	-0.002	0.011	-0.166	0.868
NETRIN1 anterior_thalamic_radiation	-0.021	0.011	-1.833	0.067
NETRIN1 cingulate_gyrus_part_of_cingulum	-0.013	0.011	-1.255	0.209
NETRIN1 parahippocampal_part_of_cingulum	-0.006	0.011	-0.526	0.599
NETRIN1 corticospinal_tract	-0.019	0.011	-1.701	0.089
NETRIN1 inferior_fronto_occipital_fasciculus	-0.019	0.012	-1.647	0.100
NETRIN1 inferior_longitudinal_fasciculus	-0.021	0.012	-1.813	0.070
NETRIN1 medial_lemniscus	-0.008	0.010	-0.735	0.462
NETRIN1 posterior_thalamic_radiation	-0.011	0.011	-0.981	0.326
NETRIN1 superior_longitudinal_fasciculus	-0.026	0.012	-2.254	0.024
NETRIN1 superior_thalamic_radiation	-0.015	0.012	-1.251	0.211
NETRIN1 uncinate_fasciculus	-0.018	0.011	-1.680	0.093
NETRIN1 bl.FA.wm.forceps_major	-0.017	0.012	-1.409	0.159
NETRIN1 bl.FA.wm.forceps_minor	-0.011	0.012	-0.934	0.351
NETRIN1 bl.FA.wm.middle_cerebellar_peduncle	-0.029	0.012	-2.333	0.020
Genomic acoustic_radiation	-0.011	0.011	-1.033	0.301
Genomic anterior_thalamic_radiation	-0.015	0.012	-1.315	0.188
Genomic cingulate_gyrus_part_of_cingulum	-0.016	0.011	-1.528	0.127
Genomic parahippocampal_part_of_cingulum	-0.019	0.011	-1.779	0.075
Genomic corticospinal_tract	-0.008	0.011	-0.666	0.505
Genomic inferior_fronto_occipital_fasciculus	-0.023	0.012	-2.008	0.045
Genomic inferior_longitudinal_fasciculus	-0.023	0.012	-1.959	0.050
Genomic medial_lemniscus	0.003	0.010	0.306	0.760
Genomic posterior_thalamic_radiation	-0.021	0.011	-1.873	0.061
Genomic superior_longitudinal_fasciculus	-0.026	0.012	-2.244	0.025
Genomic superior_thalamic_radiation	-0.010	0.012	-0.867	0.386
Genomic uncinate_fasciculus	-0.028	0.011	-2.545	0.011
Genomic bl.FA.wm.forceps_major	-0.037	0.012	-3.042	0.002
Genomic bl.FA.wm.forceps_minor	-0.031	0.012	-2.600	0.009
Genomic bl.FA.wm.middle_cerebellar_peduncle	-0.009	0.012	-0.730	0.465
PGRS THRESHOLD: 0.05				
NETRIN1 acoustic_radiation	0.009	0.011	0.819	0.413
NETRIN1 anterior_thalamic_radiation	-0.015	0.011	-1.282	0.200
NETRIN1 cingulate_gyrus_part_of_cingulum	-0.011	0.011	-1.065	0.287
NETRIN1 parahippocampal_part_of_cingulum	-0.013	0.011	-1.197	0.232
NETRIN1 corticospinal_tract	-0.003	0.011	-0.276	0.782

	Value	Std. Error	t-value	p-value
NETRIN1 inferior_fronto_occipital_fasciculus	-0.005	0.011	-0.473	0.636
NETRIN1 inferior_longitudinal_fasciculus	-0.010	0.011	-0.841	0.400
NETRIN1 medial_lemniscus	-0.005	0.010	-0.456	0.649
NETRIN1 posterior_thalamic_radiation	-0.002	0.011	-0.205	0.838
NETRIN1 superior_longitudinal_fasciculus	-0.015	0.012	-1.265	0.206
NETRIN1 superior_thalamic_radiation	-0.001	0.012	-0.049	0.961
NETRIN1 uncinate_fasciculus	-0.009	0.011	-0.876	0.381
NETRIN1 bl.FA.wm.forceps_major	-0.008	0.012	-0.644	0.520
NETRIN1 bl.FA.wm.forceps_minor	-0.005	0.012	-0.397	0.691
NETRIN1 bl.FA.wm.middle_cerebellar_peduncle	-0.018	0.012	-1.461	0.144
Genomic acoustic_radiation	-0.012	0.011	-1.151	0.250
Genomic anterior_thalamic_radiation	-0.017	0.011	-1.459	0.145
Genomic cingulate_gyrus_part_of_cingulum	-0.019	0.011	-1.739	0.082
Genomic parahippocampal_part_of_cingulum	-0.019	0.011	-1.771	0.077
Genomic corticospinal_tract	-0.012	0.011	-1.037	0.300
Genomic inferior_fronto_occipital_fasciculus	-0.026	0.012	-2.292	0.022
Genomic inferior_longitudinal_fasciculus	-0.026	0.012	-2.252	0.024
Genomic medial_lemniscus	0.000	0.010	0.037	0.970
Genomic posterior_thalamic_radiation	-0.026	0.011	-2.357	0.018
Genomic superior_longitudinal_fasciculus	-0.029	0.012	-2.500	0.012
Genomic superior_thalamic_radiation	-0.015	0.012	-1.296	0.195
Genomic uncinate_fasciculus	-0.030	0.011	-2.725	0.006
Genomic bl.FA.wm.forceps_major	-0.037	0.012	-3.083	0.002
Genomic bl.FA.wm.forceps_minor	-0.034	0.012	-2.834	0.005
Genomic bl.FA.wm.middle_cerebellar_peduncle	-0.012	0.012	-0.983	0.326
PGRS THRESHOLD: 0.1				
NETRIN1 acoustic_radiation	0.005	0.011	0.443	0.658
NETRIN1 anterior_thalamic_radiation	-0.018	0.011	-1.580	0.114
NETRIN1 cingulate_gyrus_part_of_cingulum	-0.016	0.011	-1.528	0.127
NETRIN1 parahippocampal_part_of_cingulum	-0.006	0.011	-0.580	0.562
NETRIN1 corticospinal_tract	-0.004	0.011	-0.314	0.753
NETRIN1 inferior_fronto_occipital_fasciculus	-0.013	0.011	-1.090	0.276
NETRIN1 inferior_longitudinal_fasciculus	-0.013	0.012	-1.091	0.275
NETRIN1 medial_lemniscus	-0.016	0.010	-1.569	0.117
NETRIN1 posterior_thalamic_radiation	-0.005	0.011	-0.481	0.631
NETRIN1 superior_longitudinal_fasciculus	-0.024	0.012	-2.065	0.039
NETRIN1 superior_thalamic_radiation	-0.010	0.012	-0.827	0.408
NETRIN1 uncinate_fasciculus	-0.008	0.011	-0.756	0.450
NETRIN1 bl.FA.wm.forceps_major	-0.014	0.012	-1.145	0.252
NETRIN1 bl.FA.wm.forceps_minor	-0.011	0.012	-0.934	0.350
NETRIN1 bl.FA.wm.middle_cerebellar_peduncle	-0.013	0.012	-1.049	0.294
Genomic acoustic_radiation	-0.012	0.011	-1.147	0.251
Genomic anterior_thalamic_radiation	-0.014	0.011	-1.186	0.236
Genomic cingulate_gyrus_part_of_cingulum	-0.018	0.011	-1.699	0.089

	Value	Std. Error	t-value	p-value
Genomic parahippocampal_part_of_cingulum	-0.017	0.011	-1.552	0.121
Genomic corticospinal_tract	-0.016	0.011	-1.379	0.168
Genomic inferior_fronto_occipital_fasciculus	-0.025	0.012	-2.177	0.030
Genomic inferior_longitudinal_fasciculus	-0.024	0.012	-2.121	0.034
Genomic medial_lemniscus	0.001	0.010	0.139	0.890
Genomic posterior_thalamic_radiation	-0.022	0.011	-2.002	0.045
Genomic superior_longitudinal_fasciculus	-0.026	0.012	-2.267	0.023
Genomic superior_thalamic_radiation	-0.014	0.012	-1.188	0.235
Genomic uncinate_fasciculus	-0.032	0.011	-2.915	0.004
Genomic bl.FA.wm.forceps_major	-0.033	0.012	-2.753	0.006
Genomic bl.FA.wm.forceps_minor	-0.031	0.012	-2.572	0.010
Genomic bl.FA.wm.middle_cerebellar_peduncle	-0.018	0.012	-1.465	0.143
PGRS THRESHOLD: 1				
NETRIN1 acoustic_radiation	0.003	0.011	0.294	0.769
NETRIN1 anterior_thalamic_radiation	-0.023	0.011	-2.051	0.040
NETRIN1 cingulate_gyrus_part_of_cingulum	-0.029	0.011	-2.720	0.007
NETRIN1 parahippocampal_part_of_cingulum	-0.007	0.011	-0.692	0.489
NETRIN1 corticospinal_tract	0.001	0.011	0.120	0.905
NETRIN1 inferior_fronto_occipital_fasciculus	-0.024	0.011	-2.070	0.039
NETRIN1 inferior_longitudinal_fasciculus	-0.023	0.011	-1.978	0.048
NETRIN1 medial_lemniscus	-0.008	0.010	-0.757	0.449
NETRIN1 posterior_thalamic_radiation	-0.015	0.011	-1.360	0.174
NETRIN1 superior_longitudinal_fasciculus	-0.035	0.012	-3.017	0.003
NETRIN1 superior_thalamic_radiation	-0.006	0.012	-0.517	0.605
NETRIN1 uncinate_fasciculus	-0.019	0.011	-1.799	0.072
NETRIN1 forceps_major	-0.016	0.012	-1.333	0.183
NETRIN1 forceps_minor	-0.018	0.012	-1.537	0.124
NETRIN1 middle_cerebellar_peduncle	-0.016	0.012	-1.294	0.196
Genomic acoustic_radiation	-0.013	0.011	-1.230	0.219
Genomic anterior_thalamic_radiation	-0.016	0.011	-1.386	0.166
Genomic cingulate_gyrus_part_of_cingulum	-0.021	0.011	-1.943	0.052
Genomic parahippocampal_part_of_cingulum	-0.022	0.011	-2.022	0.043
Genomic corticospinal_tract	-0.018	0.011	-1.604	0.109
Genomic inferior_fronto_occipital_fasciculus	-0.028	0.012	-2.444	0.015
Genomic inferior_longitudinal_fasciculus	-0.025	0.012	-2.135	0.033
Genomic medial_lemniscus	-0.004	0.010	-0.401	0.689
Genomic posterior_thalamic_radiation	-0.022	0.011	-1.923	0.054
Genomic superior_longitudinal_fasciculus	-0.022	0.012	-1.927	0.054
Genomic superior_thalamic_radiation	-0.014	0.012	-1.202	0.229
Genomic uncinate_fasciculus	-0.032	0.011	-2.957	0.003
Genomic forceps_major	-0.031	0.012	-2.589	0.010
Genomic forceps_minor	-0.031	0.012	-2.573	0.010
Genomic middle_cerebellar_peduncle	-0.020	0.012	-1.585	0.113

Table S7. The effect of unpruned NETRIN1- and Genomic-PRS at thresholds 0.01, 0.05, 0.1, 0.5 and 1 on tract categories (FA) (N = 6,401).

PGRS THRESHOLD: 0.01	Value	Std. Error	t-value	p-value
NETRIN1 gFA	-0.025	0.012	-2.065	0.039
NETRIN1 Association fibres	-0.024	0.012	-2.024	0.043
NETRIN1 Thalamic radiations	-0.020	0.012	-1.615	0.106
NETRIN1 Projection fibres	-0.024	0.012	-1.963	0.050
Genomic gFA	-0.029	0.012	-2.431	0.015
Genomic Association fibres	-0.031	0.012	-2.574	0.010
Genomic Thalamic radiations	-0.020	0.012	-1.685	0.092
Genomic Projection fibres	-0.021	0.012	-1.716	0.086
PGRS THRESHOLD: 0.05				
NETRIN1 gFA	-0.012	0.012	-1.030	0.303
NETRIN1 Association fibres	-0.016	0.012	-1.333	0.183
NETRIN1 Thalamic radiations	-0.007	0.012	-0.590	0.555
NETRIN1 Projection fibres	-0.007	0.012	-0.552	0.581
Genomic gFA	-0.033	0.012	-2.776	0.006
Genomic Association fibres	-0.034	0.012	-2.845	0.004
Genomic Thalamic radiations	-0.026	0.012	-2.128	0.033
Genomic Projection fibres	-0.025	0.012	-2.073	0.038
PGRS THRESHOLD: 0.1				
NETRIN1 gFA	-0.018	0.012	-1.494	0.135
NETRIN1 Association fibres	-0.020	0.012	-1.684	0.092
NETRIN1 Thalamic radiations	-0.014	0.012	-1.125	0.261
NETRIN1 Projection fibres	-0.012	0.012	-1.032	0.302
Genomic gFA	-0.032	0.012	-2.656	0.008
Genomic Association fibres	-0.032	0.012	-2.728	0.006
Genomic Thalamic radiations	-0.022	0.012	-1.820	0.069
Genomic Projection fibres	-0.026	0.012	-2.201	0.028
PGRS THRESHOLD: 1				
NETRIN1 gFA	-0.027	0.012	-2.288	0.022
NETRIN1 Association fibres	-0.034	0.012	-2.903	0.004
NETRIN1 Thalamic radiations	-0.019	0.012	-1.590	0.112
NETRIN1 Projection fibres	-0.011	0.012	-0.881	0.379
Genomic gFA	-0.034	0.012	-2.824	0.005
Genomic Association fibres	-0.035	0.012	-2.927	0.003
Genomic Thalamic radiations	-0.023	0.012	-1.863	0.062
Genomic Projection fibres	-0.029	0.012	-2.443	0.015

Table S8. The effect of unpruned NETRIN1- and Genomic-PRS at threshold 0.5 on individual white matter tracts (FA) (N = 6,420).

PGRS THRESHOLD: 0.5	Value	Std. Error	t-value	p-value
NETRIN1 acoustic_radiation	0.002	0.011	0.222	0.824
NETRIN1 anterior_thalamic_radiation	-0.021	0.012	-1.800	0.072
NETRIN1 cingulate_gyrus_part_of_cingulum	-0.024	0.011	-2.199	0.028
NETRIN1 parahippocampal_part_of_cingulum	-0.008	0.011	-0.731	0.465
NETRIN1 corticospinal_tract	0.001	0.011	0.125	0.900
NETRIN1 inferior_fronto_occipital_fasciculus	-0.022	0.012	-1.899	0.058
NETRIN1 inferior_longitudinal_fasciculus	-0.021	0.012	-1.853	0.064
NETRIN1 medial_lemniscus	-0.009	0.010	-0.826	0.409
NETRIN1 posterior_thalamic_radiation	-0.013	0.011	-1.162	0.245
NETRIN1 superior_longitudinal_fasciculus	-0.034	0.012	-2.897	0.004
NETRIN1 superior_thalamic_radiation	-0.006	0.012	-0.466	0.641
NETRIN1 uncinate_fasciculus	-0.019	0.011	-1.698	0.090
NETRIN1 forceps_major	-0.014	0.012	-1.197	0.231
NETRIN1 forceps_minor	-0.018	0.012	-1.489	0.136
NETRIN1 middle_cerebellar_peduncle	-0.016	0.012	-1.270	0.204
Genomic acoustic_radiation	-0.016	0.011	-1.464	0.143
Genomic anterior_thalamic_radiation	-0.018	0.012	-1.530	0.126
Genomic cingulate_gyrus_part_of_cingulum	-0.020	0.011	-1.859	0.063
Genomic parahippocampal_part_of_cingulum	-0.022	0.011	-2.042	0.041
Genomic corticospinal_tract	-0.022	0.012	-1.878	0.060
Genomic inferior_fronto_occipital_fasciculus	-0.030	0.012	-2.579	0.010
Genomic inferior_longitudinal_fasciculus	-0.026	0.012	-2.258	0.024
Genomic medial_lemniscus	-0.006	0.011	-0.580	0.562
Genomic posterior_thalamic_radiation	-0.025	0.011	-2.224	0.026
Genomic superior_longitudinal_fasciculus	-0.025	0.012	-2.095	0.036
Genomic superior_thalamic_radiation	-0.018	0.012	-1.487	0.137
Genomic uncinate_fasciculus	-0.034	0.011	-3.111	0.002
Genomic forceps_major	-0.034	0.012	-2.781	0.005
Genomic forceps_minor	-0.033	0.012	-2.717	0.007
Genomic middle_cerebellar_peduncle	-0.023	0.012	-1.828	0.068

Table S9. The effect of unpruned NETRIN1- and Genomic-PRS at threshold 0.5 on tract categories (FA) (N = 6,420).

PGRS THRESHOLD: 0.5	Value	Std. Error	t-value	p-value
NETRIN1 gFA	-0.002	0.001	-2.197	0.028
NETRIN1 Association fibres	-0.002	0.001	-2.762	0.006
NETRIN1 Thalamic radiations	-0.001	0.000	-1.482	0.138
NETRIN1 Projection fibres	0.000	0.001	-0.904	0.366
Genomic gFA	-0.002	0.001	-2.769	0.006
Genomic Association fibres	-0.002	0.001	-2.836	0.005
Genomic Thalamic radiations	-0.001	0.000	-1.855	0.064
Genomic Projection fibres	-0.001	0.001	-2.415	0.016

Table S10. The effect of unpruned NETRIN1- and Genomic-PRS at thresholds 0.01, 0.05, 0.1, 0.5 and 1 on individual white matter tracts (MD) (N = 6,390).

	Value	Std. Error	t-value	p-value
PGRS THRESHOLD: 0.01				
NETRIN1 acoustic_radiation	0.008	0.011	0.772	0.440
NETRIN1 anterior_thalamic_radiation	0.018	0.011	1.694	0.090
NETRIN1 cingulate_gyrus_part_of_cingulum	0.013	0.011	1.257	0.209
NETRIN1 parahippocampal_part_of_cingulum	-0.007	0.011	-0.621	0.535
NETRIN1 corticospinal_tract	0.003	0.011	0.270	0.787
NETRIN1 inferior_fronto_occipital_fasciculus	0.021	0.011	1.905	0.057
NETRIN1 inferior_longitudinal_fasciculus	0.019	0.011	1.727	0.084
NETRIN1 medial_lemniscus	0.007	0.011	0.659	0.510
NETRIN1 posterior_thalamic_radiation	0.016	0.011	1.466	0.143
NETRIN1 superior_longitudinal_fasciculus	0.023	0.011	2.046	0.041
NETRIN1 superior_thalamic_radiation	0.016	0.010	1.589	0.112
NETRIN1 uncinate_fasciculus	0.011	0.010	1.033	0.302
NETRIN1 bl.MD.wm.forceps_major	0.013	0.012	1.083	0.279
NETRIN1 bl.MD.wm.forceps_minor	0.022	0.012	1.946	0.052
NETRIN1 bl.MD.wm.middle_cerebellar_peduncle	0.003	0.012	0.239	0.811
Genomic acoustic_radiation	0.015	0.011	1.453	0.146
Genomic anterior_thalamic_radiation	0.020	0.011	1.878	0.060
Genomic cingulate_gyrus_part_of_cingulum	0.038	0.011	3.529	0.000
Genomic parahippocampal_part_of_cingulum	0.030	0.011	2.846	0.004
Genomic corticospinal_tract	0.030	0.011	2.654	0.008
Genomic inferior_fronto_occipital_fasciculus	0.032	0.011	2.879	0.004
Genomic inferior_longitudinal_fasciculus	0.029	0.011	2.618	0.009
Genomic medial_lemniscus	0.012	0.011	1.145	0.252
Genomic posterior_thalamic_radiation	0.016	0.011	1.493	0.135
Genomic superior_longitudinal_fasciculus	0.028	0.011	2.490	0.013
Genomic superior_thalamic_radiation	0.023	0.010	2.320	0.020
Genomic uncinate_fasciculus	0.033	0.010	3.148	0.002
Genomic bl.MD.wm.forceps_major	0.033	0.012	2.733	0.006
Genomic bl.MD.wm.forceps_minor	0.020	0.012	1.692	0.091
Genomic bl.MD.wm.middle_cerebellar_peduncle	0.004	0.012	0.362	0.718

	Value	Std. Error	t-value	p-value
PGRS THRESHOLD: 0.05				
NETRIN1 acoustic_radiation	-0.006	0.010	-0.561	0.575
NETRIN1 anterior_thalamic_radiation	0.015	0.011	1.426	0.154
NETRIN1 cingulate_gyrus_part_of_cingulum	0.014	0.011	1.354	0.176
NETRIN1 parahippocampal_part_of_cingulum	0.004	0.011	0.347	0.729
NETRIN1 corticospinal_tract	-0.003	0.011	-0.226	0.821
NETRIN1 inferior_fronto_occipital_fasciculus	0.015	0.011	1.303	0.193
NETRIN1 inferior_longitudinal_fasciculus	0.017	0.011	1.538	0.124
NETRIN1 medial_lemniscus	0.002	0.011	0.160	0.873
NETRIN1 posterior_thalamic_radiation	0.016	0.011	1.509	0.131
NETRIN1 superior_longitudinal_fasciculus	0.023	0.011	1.998	0.046
NETRIN1 superior_thalamic_radiation	0.014	0.010	1.420	0.156
NETRIN1 uncinate_fasciculus	0.008	0.010	0.752	0.452
NETRIN1 bl.MD.wm.forceps_major	0.014	0.012	1.172	0.241
NETRIN1 bl.MD.wm.forceps_minor	0.015	0.012	1.292	0.196
NETRIN1 bl.MD.wm.middle_cerebellar_peduncle	-0.002	0.012	-0.138	0.890
Genomic acoustic_radiation	0.021	0.011	1.959	0.050
Genomic anterior_thalamic_radiation	0.025	0.011	2.359	0.018
Genomic cingulate_gyrus_part_of_cingulum	0.040	0.011	3.734	0.000
Genomic parahippocampal_part_of_cingulum	0.033	0.011	3.108	0.002
Genomic corticospinal_tract	0.034	0.011	2.999	0.003
Genomic inferior_fronto_occipital_fasciculus	0.037	0.011	3.327	0.001
Genomic inferior_longitudinal_fasciculus	0.032	0.011	2.890	0.004
Genomic medial_lemniscus	0.012	0.011	1.091	0.275
Genomic posterior_thalamic_radiation	0.016	0.011	1.527	0.127
Genomic superior_longitudinal_fasciculus	0.032	0.011	2.819	0.005
Genomic superior_thalamic_radiation	0.028	0.010	2.812	0.005
Genomic uncinate_fasciculus	0.032	0.010	3.116	0.002
Genomic bl.MD.wm.forceps_major	0.032	0.012	2.663	0.008
Genomic bl.MD.wm.forceps_minor	0.024	0.012	2.103	0.036
Genomic bl.MD.wm.middle_cerebellar_peduncle	0.006	0.012	0.515	0.607
PGRS THRESHOLD: 0.1				
NETRIN1 acoustic_radiation	-0.005	0.010	-0.458	0.647
NETRIN1 anterior_thalamic_radiation	0.020	0.011	1.868	0.062
NETRIN1 cingulate_gyrus_part_of_cingulum	0.014	0.011	1.334	0.182
NETRIN1 parahippocampal_part_of_cingulum	-0.007	0.011	-0.710	0.478
NETRIN1 corticospinal_tract	0.002	0.011	0.204	0.838
NETRIN1 inferior_fronto_occipital_fasciculus	0.020	0.011	1.800	0.072
NETRIN1 inferior_longitudinal_fasciculus	0.020	0.011	1.832	0.067
NETRIN1 medial_lemniscus	0.011	0.011	1.018	0.309
NETRIN1 posterior_thalamic_radiation	0.018	0.011	1.638	0.102
NETRIN1 superior_longitudinal_fasciculus	0.030	0.011	2.611	0.009
NETRIN1 superior_thalamic_radiation	0.021	0.010	2.073	0.038
NETRIN1 uncinate_fasciculus	0.009	0.010	0.879	0.379
NETRIN1 forceps_major	0.017	0.012	1.407	0.159
NETRIN1 forceps_minor	0.018	0.012	1.597	0.110
NETRIN1 middle_cerebellar_peduncle	0.004	0.012	0.298	0.766

	Value	Std. Error	t-value	p-value
Genomic acoustic_radiation	0.022	0.011	2.107	0.035
Genomic anterior_thalamic_radiation	0.023	0.011	2.143	0.032
Genomic cingulate_gyrus_part_of_cingulum	0.038	0.011	3.601	0.000
Genomic parahippocampal_part_of_cingulum	0.033	0.011	3.098	0.002
Genomic corticospinal_tract	0.032	0.011	2.802	0.005
Genomic inferior_fronto_occipital_fasciculus	0.034	0.011	3.081	0.002
Genomic inferior_longitudinal_fasciculus	0.030	0.011	2.689	0.007
Genomic medial_lemniscus	0.005	0.011	0.489	0.625
Genomic posterior_thalamic_radiation	0.009	0.011	0.884	0.377
Genomic superior_longitudinal_fasciculus	0.030	0.011	2.617	0.009
Genomic superior_thalamic_radiation	0.024	0.010	2.442	0.015
Genomic uncinate_fasciculus	0.034	0.010	3.320	0.001
Genomic forceps_major	0.028	0.012	2.358	0.018
Genomic forceps_minor	0.021	0.012	1.783	0.075
Genomic middle_cerebellar_peduncle	0.008	0.012	0.666	0.505
PGRS THRESHOLD: 1				
NETRIN1 acoustic_radiation	0.004	0.010	0.347	0.729
NETRIN1 anterior_thalamic_radiation	0.028	0.011	2.669	0.008
NETRIN1 cingulate_gyrus_part_of_cingulum	0.022	0.011	2.023	0.043
NETRIN1 parahippocampal_part_of_cingulum	0.000	0.011	-0.023	0.981
NETRIN1 corticospinal_tract	0.017	0.011	1.525	0.127
NETRIN1 inferior_fronto_occipital_fasciculus	0.028	0.011	2.551	0.011
NETRIN1 inferior_longitudinal_fasciculus	0.029	0.011	2.553	0.011
NETRIN1 medial_lemniscus	0.005	0.011	0.428	0.669
NETRIN1 posterior_thalamic_radiation	0.026	0.011	2.453	0.014
NETRIN1 superior_longitudinal_fasciculus	0.033	0.011	2.953	0.003
NETRIN1 superior_thalamic_radiation	0.027	0.010	2.763	0.006
NETRIN1 uncinate_fasciculus	0.020	0.010	1.900	0.058
NETRIN1 forceps_major	0.018	0.012	1.519	0.129
NETRIN1 forceps_minor	0.021	0.012	1.791	0.073
NETRIN1 middle_cerebellar_peduncle	0.011	0.012	0.890	0.373
Genomic acoustic_radiation	0.019	0.011	1.841	0.066
Genomic anterior_thalamic_radiation	0.021	0.011	2.021	0.043
Genomic cingulate_gyrus_part_of_cingulum	0.036	0.011	3.332	0.001
Genomic parahippocampal_part_of_cingulum	0.034	0.011	3.223	0.001
Genomic corticospinal_tract	0.023	0.011	1.997	0.046
Genomic inferior_fronto_occipital_fasciculus	0.032	0.011	2.828	0.005
Genomic inferior_longitudinal_fasciculus	0.025	0.011	2.262	0.024
Genomic medial_lemniscus	0.005	0.011	0.470	0.639
Genomic posterior_thalamic_radiation	0.002	0.011	0.142	0.887
Genomic superior_longitudinal_fasciculus	0.024	0.011	2.156	0.031
Genomic superior_thalamic_radiation	0.018	0.010	1.804	0.071
Genomic uncinate_fasciculus	0.030	0.010	2.844	0.004
Genomic forceps_major	0.029	0.012	2.447	0.014
Genomic forceps_minor	0.021	0.012	1.858	0.063
Genomic middle_cerebellar_peduncle	0.012	0.012	0.965	0.335

Table S11. The effect of unpruned NETRIN1- and Genomic-PRS at thresholds 0.01, 0.05, 0.1, 0.5 and 1 on tract categories (MD) (N = 6,390).

	Value	Std. Error	t-value	p-value
PGRS THRESHOLD: 0.01				
NETRIN1 gMD	0.018	0.012	1.574	0.116
NETRIN1 Association fibres	0.013	0.012	1.086	0.277
NETRIN1 Thalamic radiations	0.019	0.011	1.781	0.075
NETRIN1 Projection fibres	0.013	0.012	1.087	0.277
Genomic gMD	0.037	0.012	3.248	0.001
Genomic Association fibres	0.043	0.012	3.707	0.000
Genomic Thalamic radiations	0.022	0.011	2.027	0.043
Genomic Projection fibres	0.026	0.012	2.180	0.029
PGRS THRESHOLD: 0.05				
NETRIN1 gMD	0.016	0.011	1.380	0.168
NETRIN1 Association fibres	0.015	0.012	1.320	0.187
NETRIN1 Thalamic radiations	0.018	0.011	1.669	0.095
NETRIN1 Projection fibres	0.004	0.012	0.322	0.748
Genomic gMD	0.041	0.011	3.607	0.000
Genomic Association fibres	0.047	0.012	4.033	0.000
Genomic Thalamic radiations	0.025	0.011	2.334	0.020
Genomic Projection fibres	0.030	0.012	2.478	0.013
PGRS THRESHOLD: 0.1				
NETRIN1 gMD	0.018	0.011	1.596	0.111
NETRIN1 Association fibres	0.013	0.012	1.106	0.269
NETRIN1 Thalamic radiations	0.022	0.011	2.055	0.040
NETRIN1 Projection fibres	0.011	0.012	0.920	0.358
Genomic gMD	0.038	0.011	3.342	0.001
Genomic Association fibres	0.046	0.012	3.934	0.000
Genomic Thalamic radiations	0.020	0.011	1.822	0.069
Genomic Projection fibres	0.029	0.012	2.391	0.017
PGRS THRESHOLD: 1				
NETRIN1 gMD	0.029	0.011	2.524	0.012
NETRIN1 Association fibres	0.023	0.012	2.014	0.044
NETRIN1 Thalamic radiations	0.031	0.011	2.944	0.003
NETRIN1 Projection fibres	0.020	0.012	1.686	0.092
Genomic gMD	0.034	0.011	2.974	0.003
Genomic Association fibres	0.043	0.012	3.666	0.000
Genomic Thalamic radiations	0.013	0.011	1.229	0.219
Genomic Projection fibres	0.030	0.012	2.494	0.013

Table S12. The effect of unpruned NETRIN1- and Genomic-PRS at threshold 0.5 on tract categories (MD) (N = 6,420).

PGRS THRESHOLD: 0.5	Value	Std. Error	t-value	p-value
NETRIN1 acoustic_radiation	0.005	0.011	0.484	0.628
NETRIN1 anterior_thalamic_radiation	0.023	0.011	2.171	0.030
NETRIN1 cingulate_gyrus_part_of_cingulum	0.019	0.011	1.682	0.093
NETRIN1 parahippocampal_part_of_cingulum	0.000	0.011	-0.004	0.997
NETRIN1 corticospinal_tract	0.014	0.012	1.232	0.218
NETRIN1 inferior_fronto_occipital_fasciculus	0.025	0.011	2.242	0.025
NETRIN1 inferior_longitudinal_fasciculus	0.027	0.011	2.377	0.017
NETRIN1 medial_lemniscus	0.003	0.011	0.288	0.774
NETRIN1 posterior_thalamic_radiation	0.024	0.011	2.213	0.027
NETRIN1 superior_longitudinal_fasciculus	0.030	0.011	2.649	0.008
NETRIN1 superior_thalamic_radiation	0.024	0.010	2.345	0.019
NETRIN1 uncinate_fasciculus	0.017	0.011	1.559	0.119
NETRIN1 forceps_major	0.019	0.012	1.599	0.110
NETRIN1 forceps_minor	0.019	0.012	1.592	0.111
NETRIN1 middle_cerebellar_peduncle	0.012	0.012	0.984	0.325
Genomic acoustic_radiation	0.010	0.011	0.949	0.342
Genomic anterior_thalamic_radiation	0.011	0.011	1.009	0.313
Genomic cingulate_gyrus_part_of_cingulum	0.021	0.011	1.852	0.064
Genomic parahippocampal_part_of_cingulum	0.027	0.011	2.485	0.013
Genomic corticospinal_tract	0.009	0.012	0.800	0.424
Genomic inferior_fronto_occipital_fasciculus	0.019	0.011	1.630	0.103
Genomic inferior_longitudinal_fasciculus	0.013	0.011	1.138	0.255
Genomic medial_lemniscus	-0.004	0.011	-0.378	0.705
Genomic posterior_thalamic_radiation	-0.006	0.011	-0.505	0.613
Genomic superior_longitudinal_fasciculus	0.013	0.012	1.113	0.266
Genomic superior_thalamic_radiation	0.007	0.011	0.653	0.514
Genomic uncinate_fasciculus	0.017	0.011	1.588	0.112
Genomic forceps_major	0.020	0.012	1.624	0.104
Genomic forceps_minor	0.012	0.012	0.982	0.326
Genomic middle_cerebellar_peduncle	0.005	0.012	0.437	0.662

Table S13. The effect of unpruned NETRIN1- and Genomic-PRS at threshold 0.5 on tract categories (MD) (N = 6,320).

PGRS THRESHOLD: 0.5	Value	Std. Error	t-value	p-value
NETRIN1 gMD	3.4E-06	1.4E-06	2.4E+00	1.6E-02
NETRIN1 Association fibres	2.0E-06	1.1E-06	1.9E+00	5.8E-02
NETRIN1 Thalamic radiations	2.2E-06	7.9E-07	2.8E+00	5.4E-03
NETRIN1 Projection fibres	1.4E-06	8.0E-07	1.8E+00	7.7E-02
Genomic gMD	4.2E-06	1.4E-06	2.9E+00	3.5E-03
Genomic Association fibres	3.9E-06	1.1E-06	3.6E+00	3.3E-04
Genomic Thalamic radiations	9.8E-07	7.9E-07	1.2E+00	2.2E-01
Genomic Projection fibres	1.9E-06	8.1E-07	2.4E+00	1.7E-02

Table S14. The effect of pruned NETRIN1- and Genomic-PRS at thresholds 0.01, 0.05, 0.1, 0.5 and 1 on individual white matter tracts (FA) (N = 6,401).

Pruned NETRIN1- and Genomic-PRS with outliers included (6,420) and outliers excluded (6,401 for FA and 6,390 for MD) at all 5 thresholds (0.01, 0.05, 0.1, 0.5, 1)

	Value	Std. Error	t-value	p-value
PGRS THRESHOLD: 0.01				
NETRIN1 acoustic_radiation	-0.004	0.011	-0.349	0.727
NETRIN1 anterior_thalamic_radiation	-0.020	0.011	-1.709	0.087
NETRIN1 cingulate_gyrus_part_of_cingulum	-0.008	0.011	-0.704	0.482
NETRIN1 parahippocampal_part_of_cingulum	-0.007	0.011	-0.641	0.521
NETRIN1 corticospinal_tract	-0.022	0.011	-1.923	0.055
NETRIN1 inferior_fronto_occipital_fasciculus	-0.024	0.012	-2.058	0.040
NETRIN1 inferior_longitudinal_fasciculus	-0.024	0.012	-2.047	0.041
NETRIN1 medial_lemniscus	-0.012	0.010	-1.131	0.258
NETRIN1 posterior_thalamic_radiation	-0.014	0.011	-1.238	0.216
NETRIN1 superior_longitudinal_fasciculus	-0.028	0.012	-2.408	0.016
NETRIN1 superior_thalamic_radiation	-0.020	0.012	-1.677	0.094
NETRIN1 uncinate_fasciculus	-0.023	0.011	-2.156	0.031
NETRIN1 bl.FA.wm.forceps_major	-0.014	0.012	-1.137	0.255
NETRIN1 bl.FA.wm.forceps_minor	-0.009	0.012	-0.784	0.433
NETRIN1 bl.FA.wm.middle_cerebellar_peduncle	-0.028	0.012	-2.257	0.024
Genomic acoustic_radiation	-0.010	0.011	-0.942	0.346
Genomic anterior_thalamic_radiation	-0.017	0.011	-1.440	0.150
Genomic cingulate_gyrus_part_of_cingulum	-0.008	0.011	-0.785	0.432
Genomic parahippocampal_part_of_cingulum	0.009	0.011	0.810	0.418
Genomic corticospinal_tract	-0.010	0.011	-0.889	0.374
Genomic inferior_fronto_occipital_fasciculus	-0.014	0.012	-1.249	0.212
Genomic inferior_longitudinal_fasciculus	-0.012	0.012	-1.023	0.306
Genomic medial_lemniscus	0.000	0.010	0.010	0.992
Genomic posterior_thalamic_radiation	-0.011	0.011	-0.965	0.335
Genomic superior_longitudinal_fasciculus	-0.013	0.012	-1.080	0.280
Genomic superior_thalamic_radiation	-0.016	0.012	-1.332	0.183
Genomic uncinate_fasciculus	-0.019	0.011	-1.793	0.073
Genomic bl.FA.wm.forceps_major	-0.013	0.012	-1.086	0.278
Genomic bl.FA.wm.forceps_minor	-0.018	0.012	-1.475	0.140
Genomic bl.FA.wm.middle_cerebellar_peduncle	0.017	0.012	1.369	0.171
PGRS THRESHOLD: 0.05				
NETRIN1 acoustic_radiation	0.008	0.011	0.770	0.441
NETRIN1 anterior_thalamic_radiation	-0.012	0.011	-1.047	0.295
NETRIN1 cingulate_gyrus_part_of_cingulum	-0.007	0.011	-0.627	0.531
NETRIN1 parahippocampal_part_of_cingulum	-0.014	0.011	-1.335	0.182
NETRIN1 corticospinal_tract	-0.002	0.011	-0.146	0.884
NETRIN1 inferior_fronto_occipital_fasciculus	-0.007	0.011	-0.590	0.555

	Value	Std. Error	t-value	p-value
NETRIN1 inferior_longitudinal_fasciculus	-0.010	0.011	-0.865	0.387
NETRIN1 medial_lemniscus	-0.006	0.010	-0.574	0.566
NETRIN1 posterior_thalamic_radiation	-0.003	0.011	-0.304	0.761
NETRIN1 superior_longitudinal_fasciculus	-0.015	0.012	-1.290	0.197
NETRIN1 superior_thalamic_radiation	-0.003	0.012	-0.275	0.783
NETRIN1 uncinate_fasciculus	-0.011	0.011	-1.030	0.303
NETRIN1 bl.FA.wm.forceps_major	-0.004	0.012	-0.292	0.770
NETRIN1 bl.FA.wm.forceps_minor	-0.002	0.012	-0.178	0.858
NETRIN1 bl.FA.wm.middle_cerebellar_peduncle	-0.015	0.012	-1.200	0.230
Genomic acoustic_radiation	-0.005	0.011	-0.462	0.644
Genomic anterior_thalamic_radiation	-0.010	0.011	-0.901	0.367
Genomic cingulate_gyrus_part_of_cingulum	-0.004	0.011	-0.350	0.726
Genomic parahippocampal_part_of_cingulum	0.001	0.011	0.103	0.918
Genomic corticospinal_tract	-0.014	0.011	-1.272	0.203
Genomic inferior_fronto_occipital_fasciculus	-0.016	0.011	-1.351	0.177
Genomic inferior_longitudinal_fasciculus	-0.015	0.011	-1.281	0.200
Genomic medial_lemniscus	-0.006	0.010	-0.569	0.569
Genomic posterior_thalamic_radiation	-0.019	0.011	-1.716	0.086
Genomic superior_longitudinal_fasciculus	-0.012	0.012	-1.076	0.282
Genomic superior_thalamic_radiation	-0.019	0.012	-1.596	0.110
Genomic uncinate_fasciculus	-0.017	0.011	-1.557	0.119
Genomic bl.FA.wm.forceps_major	-0.013	0.012	-1.093	0.275
Genomic bl.FA.wm.forceps_minor	-0.014	0.012	-1.186	0.236
Genomic bl.FA.wm.middle_cerebellar_peduncle	-0.003	0.012	-0.271	0.786
PGRS THRESHOLD: 0.1				
NETRIN1 acoustic_radiation	0.005	0.011	0.452	0.652
NETRIN1 anterior_thalamic_radiation	-0.017	0.011	-1.442	0.149
NETRIN1 cingulate_gyrus_part_of_cingulum	-0.013	0.011	-1.238	0.216
NETRIN1 parahippocampal_part_of_cingulum	-0.007	0.011	-0.681	0.496
NETRIN1 corticospinal_tract	-0.003	0.011	-0.225	0.822
NETRIN1 inferior_fronto_occipital_fasciculus	-0.016	0.012	-1.381	0.167
NETRIN1 inferior_longitudinal_fasciculus	-0.014	0.012	-1.221	0.222
NETRIN1 medial_lemniscus	-0.018	0.010	-1.730	0.084
NETRIN1 posterior_thalamic_radiation	-0.007	0.011	-0.601	0.548
NETRIN1 superior_longitudinal_fasciculus	-0.026	0.012	-2.205	0.027
NETRIN1 superior_thalamic_radiation	-0.010	0.012	-0.871	0.384
NETRIN1 uncinate_fasciculus	-0.010	0.011	-0.896	0.370
NETRIN1 forceps_major	-0.012	0.012	-1.004	0.316
NETRIN1 forceps_minor	-0.013	0.012	-1.041	0.298
NETRIN1 middle_cerebellar_peduncle	-0.011	0.012	-0.922	0.356
Genomic acoustic_radiation	0.001	0.011	0.080	0.936
Genomic anterior_thalamic_radiation	0.002	0.011	0.146	0.884
Genomic cingulate_gyrus_part_of_cingulum	-0.001	0.011	-0.075	0.940
Genomic parahippocampal_part_of_cingulum	-0.002	0.011	-0.188	0.851
Genomic corticospinal_tract	-0.015	0.011	-1.345	0.179

	Value	Std. Error	t-value	p-value
Genomic inferior_fronto_occipital_fasciculus	-0.008	0.011	-0.723	0.469
Genomic inferior_longitudinal_fasciculus	-0.009	0.012	-0.760	0.447
Genomic medial_lemniscus	-0.001	0.010	-0.131	0.896
Genomic posterior_thalamic_radiation	-0.009	0.011	-0.770	0.441
Genomic superior_longitudinal_fasciculus	-0.007	0.012	-0.580	0.562
Genomic superior_thalamic_radiation	-0.011	0.012	-0.951	0.342
Genomic uncinate_fasciculus	-0.017	0.011	-1.572	0.116
Genomic forceps_major	-0.008	0.012	-0.632	0.528
Genomic forceps_minor	-0.006	0.012	-0.518	0.605
Genomic middle_cerebellar_peduncle	-0.016	0.012	-1.281	0.200
PGRS THRESHOLD: 0.5				
NETRIN1 acoustic_radiation	0.006	0.011	0.520	0.603
NETRIN1 anterior_thalamic_radiation	-0.021	0.011	-1.811	0.070
NETRIN1 cingulate_gyrus_part_of_cingulum	-0.023	0.011	-2.201	0.028
NETRIN1 parahippocampal_part_of_cingulum	-0.006	0.011	-0.583	0.560
NETRIN1 corticospinal_tract	0.002	0.011	0.204	0.839
NETRIN1 inferior_fronto_occipital_fasciculus	-0.021	0.011	-1.824	0.068
NETRIN1 inferior_longitudinal_fasciculus	-0.021	0.012	-1.790	0.074
NETRIN1 medial_lemniscus	-0.011	0.010	-1.061	0.289
NETRIN1 posterior_thalamic_radiation	-0.011	0.011	-0.981	0.327
NETRIN1 superior_longitudinal_fasciculus	-0.035	0.012	-3.031	0.002
NETRIN1 superior_thalamic_radiation	-0.006	0.012	-0.521	0.603
NETRIN1 uncinate_fasciculus	-0.018	0.011	-1.702	0.089
NETRIN1 forceps_major	-0.009	0.012	-0.740	0.459
NETRIN1 forceps_minor	-0.013	0.012	-1.071	0.284
NETRIN1 middle_cerebellar_peduncle	-0.017	0.012	-1.363	0.173
Genomic acoustic_radiation	-0.005	0.011	-0.488	0.625
Genomic anterior_thalamic_radiation	-0.007	0.011	-0.607	0.544
Genomic cingulate_gyrus_part_of_cingulum	-0.008	0.011	-0.780	0.435
Genomic parahippocampal_part_of_cingulum	-0.013	0.011	-1.189	0.235
Genomic corticospinal_tract	-0.022	0.011	-1.926	0.054
Genomic inferior_fronto_occipital_fasciculus	-0.018	0.011	-1.581	0.114
Genomic inferior_longitudinal_fasciculus	-0.014	0.012	-1.242	0.214
Genomic medial_lemniscus	-0.011	0.010	-1.055	0.291
Genomic posterior_thalamic_radiation	-0.015	0.011	-1.346	0.178
Genomic superior_longitudinal_fasciculus	-0.012	0.012	-1.014	0.311
Genomic superior_thalamic_radiation	-0.016	0.012	-1.381	0.167
Genomic uncinate_fasciculus	-0.023	0.011	-2.172	0.030
Genomic forceps_major	-0.015	0.012	-1.270	0.204
Genomic forceps_minor	-0.014	0.012	-1.184	0.237
Genomic middle_cerebellar_peduncle	-0.016	0.012	-1.334	0.182
PGRS THRESHOLD: 1				
NETRIN1 acoustic_radiation	0.006	0.011	0.554	0.579
NETRIN1 anterior_thalamic_radiation	-0.022	0.011	-1.896	0.058
NETRIN1 cingulate_gyrus_part_of_cingulum	-0.026	0.011	-2.428	0.015

	Value	Std. Error	t-value	p-value
NETRIN1 parahippocampal_part_of_cingulum	-0.006	0.011	-0.558	0.577
NETRIN1 corticospinal_tract	-0.001	0.011	-0.057	0.954
NETRIN1 inferior_fronto_occipital_fasciculus	-0.020	0.011	-1.765	0.078
NETRIN1 inferior_longitudinal_fasciculus	-0.019	0.011	-1.629	0.103
NETRIN1 medial_lemniscus	-0.011	0.010	-1.020	0.308
NETRIN1 posterior_thalamic_radiation	-0.011	0.011	-0.991	0.322
NETRIN1 superior_longitudinal_fasciculus	-0.034	0.012	-2.959	0.003
NETRIN1 superior_thalamic_radiation	-0.007	0.012	-0.582	0.560
NETRIN1 uncinate_fasciculus	-0.018	0.011	-1.635	0.102
NETRIN1 forceps_major	-0.008	0.012	-0.678	0.497
NETRIN1 forceps_minor	-0.013	0.012	-1.116	0.264
NETRIN1 middle_cerebellar_peduncle	-0.015	0.012	-1.195	0.232
Genomic acoustic_radiation	-0.005	0.011	-0.502	0.616
Genomic anterior_thalamic_radiation	-0.009	0.011	-0.755	0.450
Genomic cingulate_gyrus_part_of_cingulum	-0.010	0.011	-0.976	0.329
Genomic parahippocampal_part_of_cingulum	-0.015	0.011	-1.373	0.170
Genomic corticospinal_tract	-0.021	0.011	-1.826	0.068
Genomic inferior_fronto_occipital_fasciculus	-0.018	0.011	-1.588	0.112
Genomic inferior_longitudinal_fasciculus	-0.014	0.011	-1.231	0.218
Genomic medial_lemniscus	-0.011	0.010	-1.044	0.296
Genomic posterior_thalamic_radiation	-0.013	0.011	-1.151	0.250
Genomic superior_longitudinal_fasciculus	-0.010	0.012	-0.848	0.396
Genomic superior_thalamic_radiation	-0.015	0.012	-1.263	0.207
Genomic uncinate_fasciculus	-0.024	0.011	-2.174	0.030
Genomic forceps_major	-0.014	0.012	-1.167	0.243
Genomic forceps_minor	-0.012	0.012	-1.017	0.309
Genomic middle_cerebellar_peduncle	-0.017	0.012	-1.344	0.179

Table S15. The effect of pruned NETRIN1- and Genomic-PRS at thresholds 0.01, 0.05, 0.1, 0.5 and 1 on tract categories (FA) (N = 6,401).

PGRS THRESHOLD: 0.01	Value	Std. Error	t-value	p-value
NETRIN1 gFA	-0.026	0.012	-2.186	0.029
NETRIN1 Association fibres	-0.025	0.012	-2.066	0.039
NETRIN1 Thalamic radiations	-0.022	0.012	-1.853	0.064
NETRIN1 Projection fibres	-0.025	0.012	-2.098	0.036
Genomic gFA	-0.015	0.012	-1.226	0.220
Genomic Association fibres	-0.013	0.012	-1.068	0.285
Genomic Thalamic radiations	-0.018	0.012	-1.488	0.137
Genomic Projection fibres	-0.009	0.012	-0.766	0.444
PGRS THRESHOLD: 0.05	Value	Std. Error	t-value	p-value
NETRIN1 gFA	-0.011	0.012	-0.943	0.346
NETRIN1 Association fibres	-0.015	0.012	-1.245	0.213
NETRIN1 Thalamic radiations	-0.008	0.012	-0.635	0.526
NETRIN1 Projection fibres	-0.004	0.012	-0.367	0.714
Genomic gFA	-0.017	0.012	-1.385	0.166
Genomic Association fibres	-0.013	0.012	-1.074	0.283
Genomic Thalamic radiations	-0.021	0.012	-1.740	0.082
Genomic Projection fibres	-0.015	0.012	-1.283	0.200
PGRS THRESHOLD: 0.1	Value	Std. Error	t-value	p-value
NETRIN1 gFA	-0.018	0.012	-1.518	0.129
NETRIN1 Association fibres	-0.020	0.012	-1.720	0.085
NETRIN1 Thalamic radiations	-0.014	0.012	-1.147	0.251
NETRIN1 Projection fibres	-0.012	0.012	-0.981	0.327
Genomic gFA	-0.010	0.012	-0.855	0.393
Genomic Association fibres	-0.008	0.012	-0.714	0.476
Genomic Thalamic radiations	-0.008	0.012	-0.666	0.505
Genomic Projection fibres	-0.013	0.012	-1.105	0.269
PGRS THRESHOLD: 0.5	Value	Std. Error	t-value	p-value
NETRIN1 gFA	-0.023	0.012	-1.966	0.049
NETRIN1 Association fibres	-0.031	0.012	-2.567	0.010
NETRIN1 Thalamic radiations	-0.016	0.012	-1.327	0.184
NETRIN1 Projection fibres	-0.008	0.012	-0.668	0.504
Genomic gFA	-0.021	0.012	-1.794	0.073
Genomic Association fibres	-0.020	0.012	-1.656	0.098
Genomic Thalamic radiations	-0.017	0.012	-1.376	0.169
Genomic Projection fibres	-0.024	0.012	-1.983	0.047
PGRS THRESHOLD: 1	Value	Std. Error	t-value	p-value
NETRIN1 gFA	-0.024	0.012	-1.991	0.047
NETRIN1 Association fibres	-0.031	0.012	-2.585	0.010
NETRIN1 Thalamic radiations	-0.017	0.012	-1.387	0.166
NETRIN1 Projection fibres	-0.009	0.012	-0.715	0.475
Genomic gFA	-0.021	0.012	-1.793	0.073
Genomic Association fibres	-0.021	0.012	-1.741	0.082
Genomic Thalamic radiations	-0.016	0.012	-1.296	0.195
Genomic Projection fibres	-0.023	0.012	-1.899	0.058

Table S16. The effect of pruned NETRIN1- and Genomic-PRS at threshold 0.5 on individual white matter tracts (FA) (N = 6,420).

PGRS THRESHOLD: 0.5	Value	Std. Error	t-value	p-value
NETRIN1 acoustic_radiation	0.002	0.011	0.198	0.843
NETRIN1 anterior_thalamic_radiation	-0.022	0.012	-1.922	0.055
NETRIN1 cingulate_gyrus_part_of_cingulum	-0.024	0.011	-2.234	0.025
NETRIN1 parahippocampal_part_of_cingulum	-0.007	0.011	-0.644	0.520
NETRIN1 corticospinal_tract	-0.001	0.011	-0.100	0.920
NETRIN1 inferior_fronto_occipital_fasciculus	-0.023	0.012	-1.957	0.050
NETRIN1 inferior_longitudinal_fasciculus	-0.022	0.012	-1.865	0.062
NETRIN1 medial_lemniscus	-0.013	0.011	-1.240	0.215
NETRIN1 posterior_thalamic_radiation	-0.013	0.011	-1.120	0.263
NETRIN1 superior_longitudinal_fasciculus	-0.035	0.012	-3.029	0.002
NETRIN1 superior_thalamic_radiation	-0.008	0.012	-0.693	0.488
NETRIN1 uncinate_fasciculus	-0.020	0.011	-1.841	0.066
NETRIN1 forceps_major	-0.011	0.012	-0.908	0.364
NETRIN1 forceps_minor	-0.016	0.012	-1.299	0.194
NETRIN1 middle_cerebellar_peduncle	-0.017	0.012	-1.360	0.174
Genomic acoustic_radiation	-0.008	0.011	-0.773	0.439
Genomic anterior_thalamic_radiation	-0.010	0.012	-0.894	0.371
Genomic cingulate_gyrus_part_of_cingulum	-0.010	0.011	-0.925	0.355
Genomic parahippocampal_part_of_cingulum	-0.014	0.011	-1.309	0.191
Genomic corticospinal_tract	-0.025	0.012	-2.154	0.031
Genomic inferior_fronto_occipital_fasciculus	-0.021	0.012	-1.758	0.079
Genomic inferior_longitudinal_fasciculus	-0.017	0.012	-1.436	0.151
Genomic medial_lemniscus	-0.015	0.011	-1.382	0.167
Genomic posterior_thalamic_radiation	-0.018	0.011	-1.586	0.113
Genomic superior_longitudinal_fasciculus	-0.014	0.012	-1.193	0.233
Genomic superior_thalamic_radiation	-0.019	0.012	-1.613	0.107
Genomic uncinate_fasciculus	-0.025	0.011	-2.284	0.022
Genomic forceps_major	-0.019	0.012	-1.521	0.128
Genomic forceps_minor	-0.017	0.012	-1.370	0.171
Genomic middle_cerebellar_peduncle	-0.019	0.012	-1.545	0.122

Table S17. The effect of unpruned NETRIN1- and Genomic-PRS at threshold 0.5 on tract categories (FA) (N = 6,420).

PGRS THRESHOLD: 0.5	Value	Std. Error	t-value	p-value
NETRIN1 gFA	-0.002	0.001	-1.966	0.049
NETRIN1 Association fibres	-0.002	0.001	-2.567	0.010
NETRIN1 Thalamic radiations	-0.001	0.000	-1.327	0.184
NETRIN1 Projection fibres	0.000	0.001	-0.668	0.504
Genomic gFA	-0.002	0.001	-1.794	0.073
Genomic Association fibres	-0.001	0.001	-1.656	0.098
Genomic Thalamic radiations	-0.001	0.000	-1.376	0.169
Genomic Projection fibres	-0.001	0.001	-1.983	0.047

Table S18. The effect of pruned NETRIN1- and Genomic-PRS at thresholds 0.01, 0.05, 0.1, 0.5 and 1 on individual white matter tracts (MD) (N = 6,390).

	Value	Std. Error	t-value	p-value
PGRS THRESHOLD: 0.01				
NETRIN1 acoustic_radiation	0.006	0.011	0.544	0.586
NETRIN1 anterior_thalamic_radiation	0.008	0.011	0.773	0.439
NETRIN1 cingulate_gyrus_part_of_cingulum	0.013	0.011	1.241	0.215
NETRIN1 parahippocampal_part_of_cingulum	-0.012	0.011	-1.116	0.264
NETRIN1 corticospinal_tract	-0.002	0.011	-0.206	0.837
NETRIN1 inferior_fronto_occipital_fasciculus	0.017	0.011	1.482	0.138
NETRIN1 inferior_longitudinal_fasciculus	0.017	0.011	1.543	0.123
NETRIN1 medial_lemniscus	0.013	0.011	1.225	0.220
NETRIN1 posterior_thalamic_radiation	0.008	0.011	0.773	0.439
NETRIN1 superior_longitudinal_fasciculus	0.018	0.011	1.556	0.120
NETRIN1 superior_thalamic_radiation	0.008	0.010	0.851	0.395
NETRIN1 uncinate_fasciculus	0.014	0.010	1.387	0.165
NETRIN1 bl.MD.wm.forceps_major	0.009	0.012	0.741	0.459
NETRIN1 bl.MD.wm.forceps_minor	0.016	0.012	1.390	0.165
NETRIN1 bl.MD.wm.middle_cerebellar_peduncle	-0.004	0.012	-0.350	0.726
Genomic acoustic_radiation	-0.004	0.011	-0.353	0.724
Genomic anterior_thalamic_radiation	0.019	0.011	1.833	0.067
Genomic cingulate_gyrus_part_of_cingulum	0.024	0.011	2.263	0.024
Genomic parahippocampal_part_of_cingulum	0.008	0.011	0.715	0.475
Genomic corticospinal_tract	0.012	0.011	1.041	0.298
Genomic inferior_fronto_occipital_fasciculus	0.019	0.011	1.711	0.087
Genomic inferior_longitudinal_fasciculus	0.008	0.011	0.758	0.449
Genomic medial_lemniscus	0.001	0.011	0.117	0.907
Genomic posterior_thalamic_radiation	-0.001	0.011	-0.100	0.920
Genomic superior_longitudinal_fasciculus	0.017	0.011	1.503	0.133
Genomic superior_thalamic_radiation	0.018	0.010	1.831	0.067
Genomic uncinate_fasciculus	0.023	0.010	2.213	0.027
Genomic bl.MD.wm.forceps_major	0.014	0.012	1.138	0.255
Genomic bl.MD.wm.forceps_minor	0.018	0.012	1.602	0.109
Genomic bl.MD.wm.middle_cerebellar_peduncle	0.010	0.012	0.821	0.411

	Value	Std. Error	t-value	p-value
PGRS THRESHOLD: 0.05				
NETRIN1 acoustic_radiation	-0.010	0.010	-0.947	0.344
NETRIN1 anterior_thalamic_radiation	0.006	0.011	0.571	0.568
NETRIN1 cingulate_gyrus_part_of_cingulum	0.014	0.011	1.273	0.203
NETRIN1 parahippocampal_part_of_cingulum	0.001	0.011	0.082	0.935
NETRIN1 corticospinal_tract	-0.006	0.011	-0.575	0.565
NETRIN1 inferior_fronto_occipital_fasciculus	0.010	0.011	0.883	0.377
NETRIN1 inferior_longitudinal_fasciculus	0.014	0.011	1.274	0.203
NETRIN1 medial_lemniscus	0.004	0.011	0.395	0.693
NETRIN1 posterior_thalamic_radiation	0.011	0.011	0.993	0.321
NETRIN1 superior_longitudinal_fasciculus	0.018	0.011	1.549	0.122
NETRIN1 superior_thalamic_radiation	0.007	0.010	0.741	0.458
NETRIN1 uncinate_fasciculus	0.008	0.010	0.780	0.435
NETRIN1 bl.MD.wm.forceps_major	0.011	0.012	0.937	0.349
NETRIN1 bl.MD.wm.forceps_minor	0.009	0.012	0.752	0.452
NETRIN1 bl.MD.wm.middle_cerebellar_peduncle	-0.007	0.012	-0.600	0.549
Genomic acoustic_radiation	0.004	0.010	0.428	0.669
Genomic anterior_thalamic_radiation	0.018	0.011	1.754	0.079
Genomic cingulate_gyrus_part_of_cingulum	0.021	0.011	1.993	0.046
Genomic parahippocampal_part_of_cingulum	0.013	0.011	1.222	0.222
Genomic corticospinal_tract	0.019	0.011	1.674	0.094
Genomic inferior_fronto_occipital_fasciculus	0.024	0.011	2.168	0.030
Genomic inferior_longitudinal_fasciculus	0.013	0.011	1.160	0.246
Genomic medial_lemniscus	0.008	0.011	0.724	0.469
Genomic posterior_thalamic_radiation	0.001	0.011	0.091	0.928
Genomic superior_longitudinal_fasciculus	0.017	0.011	1.492	0.136
Genomic superior_thalamic_radiation	0.017	0.010	1.735	0.083
Genomic uncinate_fasciculus	0.015	0.010	1.418	0.156
Genomic bl.MD.wm.forceps_major	0.016	0.012	1.298	0.194
Genomic bl.MD.wm.forceps_minor	0.020	0.012	1.703	0.089
Genomic bl.MD.wm.middle_cerebellar_peduncle	0.010	0.012	0.791	0.429
PGRS THRESHOLD: 0.1				
NETRIN1 acoustic_radiation	-0.008	0.011	-0.727	0.468
NETRIN1 anterior_thalamic_radiation	0.014	0.011	1.297	0.195
NETRIN1 cingulate_gyrus_part_of_cingulum	0.014	0.011	1.352	0.176
NETRIN1 parahippocampal_part_of_cingulum	-0.009	0.011	-0.899	0.369
NETRIN1 corticospinal_tract	-0.001	0.011	-0.071	0.943
NETRIN1 inferior_fronto_occipital_fasciculus	0.018	0.011	1.641	0.101
NETRIN1 inferior_longitudinal_fasciculus	0.020	0.011	1.774	0.076
NETRIN1 medial_lemniscus	0.015	0.011	1.377	0.169
NETRIN1 posterior_thalamic_radiation	0.014	0.011	1.334	0.182
NETRIN1 superior_longitudinal_fasciculus	0.027	0.011	2.413	0.016
NETRIN1 superior_thalamic_radiation	0.016	0.010	1.652	0.099
NETRIN1 uncinate_fasciculus	0.010	0.010	0.991	0.321
NETRIN1 forceps_major	0.014	0.012	1.205	0.228
NETRIN1 forceps_minor	0.015	0.012	1.284	0.199

	Value	Std. Error	t-value	p-value
NETRIN1 middle_cerebellar_peduncle	0.004	0.012	0.359	0.720
Genomic acoustic_radiation	0.002	0.011	0.161	0.872
Genomic anterior_thalamic_radiation	0.011	0.011	1.023	0.306
Genomic cingulate_gyrus_part_of_cingulum	0.014	0.011	1.306	0.191
Genomic parahippocampal_part_of_cingulum	0.016	0.011	1.537	0.124
Genomic corticospinal_tract	0.013	0.011	1.167	0.243
Genomic inferior_fronto_occipital_fasciculus	0.014	0.011	1.228	0.219
Genomic inferior_longitudinal_fasciculus	0.006	0.011	0.516	0.606
Genomic medial_lemniscus	-0.003	0.011	-0.323	0.747
Genomic posterior_thalamic_radiation	-0.008	0.011	-0.791	0.429
Genomic superior_longitudinal_fasciculus	0.009	0.011	0.832	0.405
Genomic superior_thalamic_radiation	0.008	0.010	0.776	0.438
Genomic uncinate_fasciculus	0.013	0.010	1.246	0.213
Genomic forceps_major	0.009	0.012	0.751	0.453
Genomic forceps_minor	0.010	0.012	0.852	0.394
Genomic middle_cerebellar_peduncle	0.009	0.012	0.712	0.476
PGRS THRESHOLD: 0.5				
NETRIN1 acoustic_radiation	-0.002	0.010	-0.187	0.852
NETRIN1 anterior_thalamic_radiation	0.020	0.011	1.854	0.064
NETRIN1 cingulate_gyrus_part_of_cingulum	0.017	0.011	1.586	0.113
NETRIN1 parahippocampal_part_of_cingulum	-0.009	0.011	-0.813	0.416
NETRIN1 corticospinal_tract	0.008	0.011	0.694	0.488
NETRIN1 inferior_fronto_occipital_fasciculus	0.025	0.011	2.193	0.028
NETRIN1 inferior_longitudinal_fasciculus	0.025	0.011	2.221	0.026
NETRIN1 medial_lemniscus	0.004	0.011	0.338	0.736
NETRIN1 posterior_thalamic_radiation	0.020	0.011	1.878	0.060
NETRIN1 superior_longitudinal_fasciculus	0.030	0.011	2.603	0.009
NETRIN1 superior_thalamic_radiation	0.020	0.010	2.051	0.040
NETRIN1 uncinate_fasciculus	0.015	0.010	1.421	0.155
NETRIN1 forceps_major	0.014	0.012	1.181	0.237
NETRIN1 forceps_minor	0.014	0.012	1.202	0.229
NETRIN1 middle_cerebellar_peduncle	0.009	0.012	0.759	0.448
Genomic acoustic_radiation	-0.002	0.010	-0.177	0.860
Genomic anterior_thalamic_radiation	0.013	0.011	1.216	0.224
Genomic cingulate_gyrus_part_of_cingulum	0.014	0.011	1.359	0.174
Genomic parahippocampal_part_of_cingulum	0.018	0.011	1.678	0.093
Genomic corticospinal_tract	0.002	0.011	0.205	0.838
Genomic inferior_fronto_occipital_fasciculus	0.014	0.011	1.238	0.216
Genomic inferior_longitudinal_fasciculus	0.006	0.011	0.495	0.621
Genomic medial_lemniscus	-0.002	0.011	-0.204	0.839
Genomic posterior_thalamic_radiation	-0.010	0.011	-0.972	0.331
Genomic superior_longitudinal_fasciculus	0.008	0.011	0.667	0.505
Genomic superior_thalamic_radiation	0.006	0.010	0.582	0.561
Genomic uncinate_fasciculus	0.010	0.010	0.960	0.337
Genomic forceps_major	0.014	0.012	1.202	0.230
Genomic forceps_minor	0.016	0.012	1.425	0.154
Genomic middle_cerebellar_peduncle	0.011	0.012	0.870	0.384

	Value	Std. Error	t-value	p-value
PGRS THRESHOLD: 1				
NETRIN1 acoustic_radiation	-0.003	0.010	-0.333	0.739
NETRIN1 anterior_thalamic_radiation	0.022	0.011	2.070	0.039
NETRIN1 cingulate_gyrus_part_of_cingulum	0.018	0.011	1.698	0.089
NETRIN1 parahippocampal_part_of_cingulum	-0.006	0.011	-0.608	0.543
NETRIN1 corticospinal_tract	0.009	0.011	0.789	0.430
NETRIN1 inferior_fronto_occipital_fasciculus	0.024	0.011	2.176	0.030
NETRIN1 inferior_longitudinal_fasciculus	0.023	0.011	2.018	0.044
NETRIN1 medial_lemniscus	0.004	0.011	0.355	0.723
NETRIN1 posterior_thalamic_radiation	0.020	0.011	1.875	0.061
NETRIN1 superior_longitudinal_fasciculus	0.029	0.011	2.576	0.010
NETRIN1 superior_thalamic_radiation	0.021	0.010	2.132	0.033
NETRIN1 uncinate_fasciculus	0.016	0.010	1.562	0.118
NETRIN1 forceps_major	0.013	0.012	1.067	0.286
NETRIN1 forceps_minor	0.016	0.012	1.403	0.161
NETRIN1 middle_cerebellar_peduncle	0.008	0.012	0.664	0.507
Genomic acoustic_radiation	-0.003	0.010	-0.318	0.750
Genomic anterior_thalamic_radiation	0.013	0.011	1.202	0.229
Genomic cingulate_gyrus_part_of_cingulum	0.014	0.011	1.292	0.196
Genomic parahippocampal_part_of_cingulum	0.019	0.011	1.780	0.075
Genomic corticospinal_tract	0.003	0.011	0.258	0.796
Genomic inferior_fronto_occipital_fasciculus	0.013	0.011	1.167	0.243
Genomic inferior_longitudinal_fasciculus	0.005	0.011	0.471	0.638
Genomic medial_lemniscus	-0.004	0.011	-0.396	0.692
Genomic posterior_thalamic_radiation	-0.013	0.011	-1.172	0.241
Genomic superior_longitudinal_fasciculus	0.006	0.011	0.549	0.583
Genomic superior_thalamic_radiation	0.005	0.010	0.464	0.643
Genomic uncinate_fasciculus	0.010	0.010	0.983	0.326
Genomic forceps_major	0.015	0.012	1.282	0.200
Genomic forceps_minor	0.012	0.012	1.044	0.296
Genomic middle_cerebellar_peduncle	0.012	0.012	1.006	0.314

Table S19. The effect of pruned NETRIN1- and Genomic-PRS at thresholds 0.01, 0.05, 0.1, 0.5 and 1 on tract categories (MD) (N = 6,390).

	Value	Std. Error	t-value	p-value
PGRS THRESHOLD: 0.01				
NETRIN1 gMD	0.011	0.012	0.998	0.318
NETRIN1 Association fibres	0.009	0.012	0.737	0.461
NETRIN1 Thalamic radiations	0.010	0.011	0.896	0.370
NETRIN1 Projection fibres	0.005	0.012	0.429	0.668
Genomic gMD	0.018	0.011	1.546	0.122
Genomic Association fibres	0.020	0.012	1.690	0.091
Genomic Thalamic radiations	0.011	0.011	1.041	0.298
Genomic Projection fibres	0.015	0.012	1.205	0.228
PGRS THRESHOLD: 0.05				
NETRIN1 gMD	0.010	0.011	0.844	0.399
NETRIN1 Association fibres	0.012	0.012	0.995	0.320
NETRIN1 Thalamic radiations	0.010	0.011	0.916	0.360
NETRIN1 Projection fibres	-0.003	0.012	-0.268	0.789
Genomic gMD	0.021	0.011	1.798	0.072
Genomic Association fibres	0.022	0.012	1.913	0.056
Genomic Thalamic radiations	0.012	0.011	1.091	0.276
Genomic Projection fibres	0.019	0.012	1.595	0.111
PGRS THRESHOLD: 0.1				
NETRIN1 gMD	0.015	0.011	1.327	0.184
NETRIN1 Association fibres	0.011	0.012	0.970	0.332
NETRIN1 Thalamic radiations	0.017	0.011	1.583	0.114
NETRIN1 Projection fibres	0.010	0.012	0.796	0.426
Genomic gMD	0.012	0.011	1.064	0.287
Genomic Association fibres	0.018	0.012	1.539	0.124
Genomic Thalamic radiations	0.001	0.011	0.120	0.904
Genomic Projection fibres	0.012	0.012	1.010	0.312
PGRS THRESHOLD: 0.5				
NETRIN1 gMD	0.020	0.011	1.783	0.075
NETRIN1 Association fibres	0.015	0.012	1.328	0.184
NETRIN1 Thalamic radiations	0.023	0.011	2.169	0.030
NETRIN1 Projection fibres	0.014	0.012	1.171	0.242
Genomic gMD	0.012	0.011	1.045	0.296
Genomic Association fibres	0.018	0.012	1.526	0.127
Genomic Thalamic radiations	0.001	0.011	0.050	0.960
Genomic Projection fibres	0.014	0.012	1.174	0.240
PGRS THRESHOLD: 1				
NETRIN1 gMD	0.021	0.011	1.829	0.068
NETRIN1 Association fibres	0.016	0.012	1.412	0.158
NETRIN1 Thalamic radiations	0.024	0.011	2.263	0.024
NETRIN1 Projection fibres	0.013	0.012	1.060	0.289
Genomic gMD	0.011	0.011	0.979	0.328
Genomic Association fibres	0.018	0.012	1.533	0.125
Genomic Thalamic radiations	-0.001	0.011	-0.091	0.928
Genomic Projection fibres	0.015	0.012	1.206	0.228

Table S20. The effect of unpruned NETRIN1- and Genomic-PRS at threshold 0.5 on individual white matter tracts (MD) (N = 6,420).

PGRS THRESHOLD: 0.5	Value	Std. Error	t-value	p-value
NETRIN1 acoustic_radiation	-0.002	0.011	-0.223	0.824
NETRIN1 anterior_thalamic_radiation	0.015	0.011	1.434	0.151
NETRIN1 cingulate_gyrus_part_of_cingulum	0.013	0.011	1.153	0.249
NETRIN1 parahippocampal_part_of_cingulum	-0.008	0.011	-0.762	0.446
NETRIN1 corticospinal_tract	0.004	0.012	0.341	0.733
NETRIN1 inferior_fronto_occipital_fasciculus	0.020	0.011	1.764	0.078
NETRIN1 inferior_longitudinal_fasciculus	0.020	0.011	1.779	0.075
NETRIN1 medial_lemniscus	0.001	0.011	0.059	0.953
NETRIN1 posterior_thalamic_radiation	0.017	0.011	1.588	0.112
NETRIN1 superior_longitudinal_fasciculus	0.023	0.011	2.036	0.042
NETRIN1 superior_thalamic_radiation	0.015	0.010	1.456	0.145
NETRIN1 uncinate_fasciculus	0.012	0.011	1.102	0.271
NETRIN1 forceps_major	0.014	0.012	1.146	0.252
NETRIN1 forceps_minor	0.011	0.012	0.949	0.342
NETRIN1 middle_cerebellar_peduncle	0.007	0.012	0.573	0.567
Genomic acoustic_radiation	-0.009	0.011	-0.817	0.414
Genomic anterior_thalamic_radiation	0.003	0.011	0.261	0.794
Genomic cingulate_gyrus_part_of_cingulum	0.003	0.011	0.234	0.815
Genomic parahippocampal_part_of_cingulum	0.010	0.011	0.980	0.327
Genomic corticospinal_tract	-0.008	0.012	-0.656	0.512
Genomic inferior_fronto_occipital_fasciculus	0.002	0.011	0.164	0.870
Genomic inferior_longitudinal_fasciculus	-0.005	0.011	-0.427	0.669
Genomic medial_lemniscus	-0.009	0.011	-0.790	0.430
Genomic posterior_thalamic_radiation	-0.018	0.011	-1.651	0.099
Genomic superior_longitudinal_fasciculus	-0.002	0.012	-0.187	0.851
Genomic superior_thalamic_radiation	-0.004	0.011	-0.335	0.738
Genomic uncinate_fasciculus	0.000	0.011	-0.035	0.972
Genomic forceps_major	0.005	0.012	0.440	0.660
Genomic forceps_minor	0.004	0.012	0.352	0.725
Genomic middle_cerebellar_peduncle	0.006	0.012	0.455	0.649

Table S21. The effect of unpruned NETRIN1- and Genomic-PRS at threshold 0.5 on tract categories (MD) (N = 6,420).

PGRS THRESHOLD: 0.5	Value	Std. Error	t-value	p-value
NETRIN1 gMD	2.53E-06	1.42E-06	1.78E+00	7.47E-02
NETRIN1 Association fibres	1.43E-06	1.08E-06	1.33E+00	1.84E-01
NETRIN1 Thalamic radiations	1.71E-06	7.87E-07	2.17E+00	3.01E-02
NETRIN1 Projection fibres	9.36E-07	8.00E-07	1.17E+00	2.42E-01
Genomic gMD	1.50E-06	1.43E-06	1.04E+00	2.96E-01
Genomic Association fibres	1.66E-06	1.09E-06	1.53E+00	1.27E-01
Genomic Thalamic radiations	3.99E-08	7.93E-07	5.02E-02	9.60E-01
Genomic Projection fibres	9.47E-07	8.06E-07	1.17E+00	2.40E-01

Results depicted in tables S6 – S21 indicate secondary analyses which complement our primary analyses. These consist firstly of the effect unpruned NETRIN1- and genomic-PRS on FA and MD values, conducted on both the full dataset (N = 6,420) and the dataset with excluded outliers (N = 6,401 and 6,390 for FA and MD, respectively). Secondly, we also investigated the effect of pruned NETRIN1- and genomic-PRS on FA and MD values, again conducted on both the full dataset and dataset with excluded outliers. The analyses consist of PRS at all five p-value thresholds (0.01, 0.05, 0.1, 0.5 and 1). A similar pattern is observed for significance in white matter tracts associated with both PRS lists across PRS thresholds within the dataset with outliers removed. Some tracts remain significant within the full sample dataset as compared to the sample with outliers removed at PRS threshold 0.5, however there is a trend towards more significant results when outliers are removed. Please refer to tables S8-S9, S12-S13, S16-S17 and S20-S21 for an account of results at PRS threshold 0.5 within the full dataset, which are directly comparable to the primary results depicted in the manuscript (PRS threshold 0.5 with outliers removed).

White matter tracts significantly associated with both NETRIN1-PRS and genomic-PRS

Fractional anisotropy

Tract categories

Significantly lower FA values in association fibres were found for both NETRIN1-PRS (β = -0.032, $p_{corrected}$ = 0.023) and genomic-PRS (β = -0.033, $p_{corrected}$ = 0.011).

Mean diffusivity

Global measures

Significantly higher gMD was associated with both NETRIN1-PRS (β = 0.027, $p_{corrected}$ = 0.031) and genomic-PRS (β = 0.033, $p_{corrected}$ = 0.006).

Individual white matter tracts

Significantly higher MD in the inferior fronto-occipital fasciculus was found for both NETRIN1-PRS ($\beta = 0.027$, $p_{corrected} = 0.046$) and genomic-PRS ($\beta = 0.031$, $p_{corrected} = 0.018$).

Data analysis code – example

```
############## NETRIN1 & Genomic-PRS analysis ##################

# PREP DATA - READ IN FILES & APPLY PCA FOR REMOVING OUTLIERS

library(dplyr)
Library(nlme)

# Read in PRS lists and imaging data

# OUTLIER EXCLUSION - FA (apply PCA on all regions in original dataset)

IM.measure = 'FA.wm' # for FA white matter tracts
dat_sub=targetdata[,c(1,grep(IM.measure,colnames(targetdata)),865:ncol(targetdata))]
dat_sub=dat_sub[complete.cases(dat_sub[,grep(IM.measure,colnames(dat_sub))]),]

targetdata=dat_sub
pca_data = targetdata[,c(grep(IM.measure,colnames(targetdata)))]
pca_fit <- princomp((-1*pca_data), scores = TRUE)
pca_score <- pca_fit$scores[,1]
dat_outlier_clear=targetdata[(scale(pca_score)> -3)&(scale(pca_score)<3),]

############

# Read in dataset containing unrelated British individual to exclude related individuals

#Merge NETRIN1 PGRS with target imaging data

#UKB_merge1 = merge(UKB_subset,NETRIN1_pgrs,by="f.eid",all.x=TRUE)

# Read in: MRI position covariates, descriptive statistics (age, sex, etc.), principal
components, genotype array

# Merge imaging with all covariates that were read in above

# Example: Merge baseline with UKB_merge1 (containing PRS and imaging data)

baseline = data.frame(baseline[,1:2])
colnames(baseline)[2] <-"sex"
UKB_merge4 = merge(UKB_merge1,baseline,by="f.eid",all.x=TRUE)

# And do the same for all other covariates
```

```
# SCALE DEPENDENT AND INDEPENDENT VARIABLES BEFORE PUTTING THEM
IN LONG FORMAT

UKB_final[,c(2:28,31:40)] <- scale(UKB_final[,c(2:28,31:40)]) #2:28 are the white matter
tracts, while 31:40 are the NETRIN1 PGRS
#######   Prep long format data   ###### output: data_long

# settings; change colnames of non-imaging data and imaging data according to the dataset
you are using - with or without outliers

targetdata = UKB_final
cols_nonimg = colnames(targetdata)[29:ncol(targetdata)]  # colnames of non-imaging data
dat_colnames = colnames(targetdata)[2:28]  # colnames of imaging data
measures = c('FA.wm')

#==============================================================
====#
source('/path/to/script/long_format.R')
dat_long <- long_format(targetdata,cols_nonimg,cols_img,measures)

# set the data in regression format
# the sequence of data from left to right:
# f.eid || factors || dependent variables || covariates
# Order the column numbers below according to the variables above: f.eid first, then factors
(PGRS lists),
# then dependent variables (DTI variables in this case), and then covariates (age, sex, 15 PCs,
genotype array, hemisphere)
# (factors=the last input of an equation)

targetdata_long = dat_long[,c(1,16:25,2:13,15,26,28:30,27,31:34,35:49,50)]     # long-format
data
#This includes: f.eid, 10 PGRS lists, 12 bilateral DTI measures, MRI age calculated,
#sex, pos_covar, array, PCs & hemi
targetdata_long = targetdata_long[complete.cases(targetdata_long),] #complete cases for all
variables

targetdata_short = UKB_final[,c(1,31:40,2:28,30,41,43:45,42,46:49,50:64)]    # short-format
data for outliers excluded
targetdata_short = targetdata_short[complete.cases(targetdata_short),] #complete cases for all
variables

# REGRESSION ANALYSIS
# FA - 0.5; use targetdata_long and targetdata_short (depending on white matter tract) for
analysis

WM_list <- colnames(targetdata_long)[2:13] # select all WM tracts to run regression
no_WM_list <- length(WM_list) # assign length of columns

# create a named list to hold the fitted models
fitlist <- as.list(1:no_WM_list)
```

```
names(fitlist) <- WM_list

for(i in WM_list){
  # create temporary data matrix and model formula
  tmp <- targetdata_long[, c(i,"sex","MRI_age.calculated","pc1","pc2","pc3","pc4",
                             "pc5","pc6","pc7","pc8","pc9","pc10","pc11","pc12","pc13","pc14","pc15
","genotyping.array",
                             "pos.x", "pos.y",
"pos.z","NETRIN1_WG_pT_0.5","NETRIN1_pathway_pT_0.5","f.eid")]
  fml <- as.formula( paste(i, "~",
                           paste(c("sex","I(MRI_age.calculated^2)","MRI_age.calculated","pc1","pc2",
"pc3","pc4","pc5","pc6","pc7","pc8","pc9",
                                   "pc10","pc11","pc12","pc13","pc14","pc15","genotyping.array","pos.x
", "pos.y",
                                   "pos.z",”hemi”,"NETRIN1_WG_pT_0.5","NETRIN1_pathway_pT_0.
5"), collapse="+") ) )
  # assign fit to list by name
  fitlist[[i]] <- lme(fml, random=~1|f.eid,na.action=na.exclude,control=lmeControl(opt =
"optim"), data=tmp)
}

FA_0.5 <- lapply(fitlist, summary)

# FDR correction – conducted separately for NETRIN1 and Genomic-PRS p-values

# INDIVIDUAL WHITE MATTER TRACTS

FA_Pvalues_categories_ind_tracts$p.corrected_fdr =
  p.adjust(FA_Pvalues_categories_ind_tracts$V2,
           method = "fdr")

write.table(FA_Pvalues_categories_ind_tracts, "FA_ind_tract_FDR_Pvalue",sep="\t")

# TRACT CATEGORIES

FA_Pvalues_categories_gFA$p.corrected_fdr =
  p.adjust(FA_Pvalues_categories_gFA$V2,
           method = "fdr")

write.table(FA_Pvalues_categories_gFA, " FA_categories_FDR_Pvalue",sep="\t")
```

The members of the Major Depressive Disorder Working Group of the Psychiatric Genomics Consortium are:

Naomi R Wray 1, 2

Stephan Ripke 3, 4, 5

Manuel Mattheisen 6, 7, 8, 9

Maciej Trzaskowski* 1

Enda M Byrne 1

Abdel Abdellaoui 10

Mark J Adams 11

Esben Agerbo 9, 12, 13

Tracy M Air 14

Till F M Andlauer 15, 16

Silviu-Alin Bacanu 17

Marie Bækvad-Hansen 9, 18

Aartjan T F Beekman 19

Tim B Bigdeli 17, 20

Elisabeth B Binder 15, 21

Douglas H R Blackwood 11

Julien Bryois 22

Henriette N Buttenschøn 8, 9, 23

Jonas Bybjerg-Grauholm 9, 18

Na Cai 24, 25

Enrique Castelao 26

Jane Hvarregaard Christensen 7, 8, 9

Toni-Kim Clarke 11

Jonathan R I Coleman 27

Lucía Colodro-Conde 28

Baptiste Couvy-Duchesne 29, 30

Nick Craddock 31

Gregory E Crawford 32, 33

Gail Davies 34

Ian J Deary 34

Franziska Degenhardt 35, 36

Eske M Derks 28

Nese Direk 37, 38

Conor V Dolan 10

Erin C Dunn 39, 40, 41

Thalia C Eley 27

Valentina Escott-Price 42

Farnush Farhadi Hassan Kiadeh 43

Hilary K Finucane 44, 45

Andreas J Forstner 35, 36, 46, 47

Josef Frank 48

Héléna A Gaspar 27

Michael Gill 49

Fernando S Goes 50

Scott D Gordon 51

Jakob Grove 7, 8, 9, 52

Lynsey S Hall 11, 53

Christine Søholm Hansen 9, 18

Thomas F Hansen 54, 55, 56

Stefan Herms 35, 36, 47

Ian B Hickie 57

Per Hoffmann 35, 36, 47

Georg Homuth 58

Carsten Horn 59

Jouke-Jan Hottenga 10

David M Hougaard 9, 18

Marcus Ising 60

Rick Jansen 19, 19

Eric Jorgenson 61

James A Knowles 62

Isaac S Kohane 63, 64, 65

Julia Kraft 4

Warren W. Kretzschmar 66

Jesper Krogh 67

Zoltán Kutalik 68, 69

Yihan Li 66

Penelope A Lind 28

Donald J MacIntyre 70, 71

Dean F MacKinnon 50

Robert M Maier 2

Wolfgang Maier 72

Jonathan Marchini 73

Hamdi Mbarek 10

Patrick McGrath 74

Peter McGuffin 27

Sarah E Medland 28

Divya Mehta 2, 75

Christel M Middeldorp 10, 76, 77

Evelin Mihailov 78

Yuri Milaneschi 19, 19

Lili Milani 78

Francis M Mondimore 50

Grant W Montgomery 1

Sara Mostafavi 79, 80

Niamh Mullins 27

Matthias Nauck 81, 82

Bernard Ng 80

Michel G Nivard 10

Dale R Nyholt 83

Paul F O'Reilly 27

Hogni Oskarsson 84

Michael J Owen 85

Jodie N Painter 28

Carsten Bøcker Pedersen 9, 12, 13

Marianne Giørtz Pedersen 9, 12, 13

Roseann E. Peterson 17, 86

Erik Pettersson 22

Wouter J Peyrot 19

Giorgio Pistis 26

Danielle Posthuma 87, 88

Jorge A Quiroz 89

Per Qvist 7, 8, 9

John P Rice 90

Brien P. Riley 17

Margarita Rivera 27, 91

Saira Saeed Mirza 37

Robert Schoevers 92

Eva C Schulte 93, 94
Ling Shen 61
Jianxin Shi 95
Stanley I Shyn 96
Engilbert Sigurdsson 97
Grant C B Sinnamon 98
Johannes H Smit 19
Daniel J Smith 99
Hreinn Stefansson 100
Stacy Steinberg 100
Fabian Streit 48
Jana Strohmaier 48
Katherine E Tansey 101
Henning Teismann 102
Alexander Teumer 103
Wesley Thompson 9, 55, 104, 105
Pippa A Thomson 106
Thorgeir E Thorgeirsson 100
Matthew Traylor 107
Jens Treutlein 48
Vassily Trubetskoy 4
André G Uitterlinden 108
Daniel Umbricht 109
Sandra Van der Auwera 110
Albert M van Hemert 111
Alexander Viktorin 22
Peter M Visscher 1, 2
Yunpeng Wang 9, 55, 105
Bradley T. Webb 112
Shantel Marie Weinsheimer 9, 55
Jürgen Wellmann 102
Gonneke Willemsen 10
Stephanie H Witt 48
Yang Wu 1
Hualin S Xi 113
Jian Yang 2, 114
Futao Zhang 1
Volker Arolt 115
Bernhard T Baune 14
Klaus Berger 102
Dorret I Boomsma 10
Sven Cichon 35, 47, 116, 117
Udo Dannlowski 115
EJC de Geus 10, 118
J Raymond DePaulo 50
Enrico Domenici 119
Katharina Domschke 120
Tõnu Esko 5, 78
Hans J Grabe 110
Steven P Hamilton 121
Caroline Hayward 122
Andrew C Heath 90
Kenneth S Kendler 17
Stefan Kloiber 60, 123, 124
Glyn Lewis 125
Qingqin S Li 126
Susanne Lucae 60
Pamela AF Madden 90
Patrik K Magnusson 22
Nicholas G Martin 51
Andrew M McIntosh 11, 34
Andres Metspalu 78, 127
Ole Mors 9, 128
Preben Bo Mortensen 8, 9, 12, 13
Bertram Müller-Myhsok 15, 16, 129
Merete Nordentoft 9, 130
Markus M Nöthen 35, 36
Michael C O'Donovan 85
Sara A Paciga 131
Nancy L Pedersen 22
Brenda WJH Penninx 19
Roy H Perlis 39, 132
David J Porteous 106
James B Potash 133
Martin Preisig 26
Marcella Rietschel 48
Catherine Schaefer 61
Thomas G Schulze 48, 94, 134, 135, 136
Jordan W Smoller 39, 40, 41
Kari Stefansson 100, 137
Henning Tiemeier 37, 138, 139
Rudolf Uher 140
Henry Völzke 103
Myrna M Weissman 74, 141
Thomas Werge 9, 55, 142
Cathryn M Lewis 27, 143
Douglas F Levinson 144
Gerome Breen 27, 145
Anders D Børglum 7, 8, 9
Patrick F Sullivan 22, 146, 147,

1, Institute for Molecular Bioscience, The University of Queensland, Brisbane, QLD, AU

2, Queensland Brain Institute, The University of Queensland, Brisbane, QLD, AU

3, Analytic and Translational Genetics Unit, Massachusetts General Hospital, Boston, MA, US

4, Department of Psychiatry and Psychotherapy, Universitätsmedizin Berlin Campus Charité Mitte, Berlin, DE

5, Medical and Population Genetics, Broad Institute, Cambridge, MA, US

6, Centre for Psychiatry Research, Department of Clinical Neuroscience, Karolinska Institutet, Stockholm, SE

7, Department of Biomedicine, Aarhus University, Aarhus, DK

8, iSEQ, Centre for Integrative Sequencing, Aarhus University, Aarhus, DK

9, iPSYCH, The Lundbeck Foundation Initiative for Integrative Psychiatric Research,, DK

10, Dept of Biological Psychology & EMGO+ Institute for Health and Care Research, Vrije Universiteit Amsterdam, Amsterdam, NL

11, Division of Psychiatry, University of Edinburgh, Edinburgh, GB

12, Centre for Integrated Register-based Research, Aarhus University, Aarhus, DK

13, National Centre for Register-Based Research, Aarhus University, Aarhus, DK

14, Discipline of Psychiatry, University of Adelaide, Adelaide, SA, AU

15, Department of Translational Research in Psychiatry, Max Planck Institute of Psychiatry, Munich, DE

16, Munich Cluster for Systems Neurology (SyNergy), Munich, DE

17, Department of Psychiatry, Virginia Commonwealth University, Richmond, VA, US

18, Center for Neonatal Screening, Department for Congenital Disorders, Statens Serum Institut, Copenhagen, DK

19, Department of Psychiatry, Vrije Universiteit Medical Center and GGZ inGeest, Amsterdam, NL

20, Virginia Institute for Psychiatric and Behavior Genetics, Richmond, VA, US

21, Department of Psychiatry and Behavioral Sciences, Emory University School of Medicine, Atlanta, GA, US

22, Department of Medical Epidemiology and Biostatistics, Karolinska Institutet, Stockholm, SE

23, Department of Clinical Medicine, Translational Neuropsychiatry Unit, Aarhus University, Aarhus, DK

24, Human Genetics, Wellcome Trust Sanger Institute, Cambridge, GB

25, Statistical genomics and systems genetics, European Bioinformatics Institute (EMBL-EBI), Cambridge, GB

26, Department of Psychiatry, University Hospital of Lausanne, Prilly, Vaud, CH

27, MRC Social Genetic and Developmental Psychiatry Centre, King's College London, London, GB

28, Genetics and Computational Biology, QIMR Berghofer Medical Research Institute, Herston, QLD, AU

29, Centre for Advanced Imaging, The University of Queensland, Saint Lucia, QLD, AU

30, Queensland Brain Institute, The University of Queensland, Saint Lucia, QLD, AU

31, Psychological Medicine, Cardiff University, Cardiff, GB

32, Center for Genomic and Computational Biology, Duke University, Durham, NC, US

33, Department of Pediatrics, Division of Medical Genetics, Duke University, Durham, NC, US

34, Centre for Cognitive Ageing and Cognitive Epidemiology, University of Edinburgh, Edinburgh, GB

35, Institute of Human Genetics, University of Bonn, Bonn, DE

36, Life&Brain Center, Department of Genomics, University of Bonn, Bonn, DE

37, Epidemiology, Erasmus MC, Rotterdam, Zuid-Holland, NL

38, Psychiatry, Dokuz Eylul University School Of Medicine, Izmir, TR

39, Department of Psychiatry, Massachusetts General Hospital, Boston, MA, US

40, Psychiatric and Neurodevelopmental Genetics Unit (PNGU), Massachusetts General Hospital, Boston, MA, US

41, Stanley Center for Psychiatric Research, Broad Institute, Cambridge, MA, US

42, Neuroscience and Mental Health, Cardiff University, Cardiff, GB

43, Bioinformatics, University of British Columbia, Vancouver, BC, CA

44, Department of Epidemiology, Harvard T.H. Chan School of Public Health, Boston, MA, US

45, Department of Mathematics, Massachusetts Institute of Technology, Cambridge, MA, US

46, Department of Psychiatry (UPK), University of Basel, Basel, CH

47, Human Genomics Research Group, Department of Biomedicine, University of Basel, Basel, CH

48, Department of Genetic Epidemiology in Psychiatry, Central Institute of Mental Health, Medical Faculty Mannheim, Heidelberg University, Mannheim, Baden-Württemberg, DE

49, Department of Psychiatry, Trinity College Dublin, Dublin, IE

50, Psychiatry & Behavioral Sciences, Johns Hopkins University, Baltimore, MD, US

51, Genetics and Computational Biology, QIMR Berghofer Medical Research Institute, Brisbane, QLD, AU

52, Bioinformatics Research Centre, Aarhus University, Aarhus, DK

53, Institute of Genetic Medicine, Newcastle University, Newcastle upon Tyne, GB

54, Danish Headache Centre, Department of Neurology, Rigshospitalet, Glostrup, DK

55, Institute of Biological Psychiatry, Mental Health Center Sct. Hans, Mental Health Services Capital Region of Denmark, Copenhagen, DK

56, iPSYCH, The Lundbeck Foundation Initiative for Psychiatric Research, Copenhagen, DK

57, Brain and Mind Centre, University of Sydney, Sydney, NSW, AU

58, Interfaculty Institute for Genetics and Functional Genomics, Department of Functional Genomics, University Medicine and Ernst Moritz Arndt University Greifswald, Greifswald, Mecklenburg-Vorpommern, DE

59, Roche Pharmaceutical Research and Early Development, Pharmaceutical Sciences, Roche Innovation Center Basel, F. Hoffmann-La Roche Ltd, Basel, CH

60, Max Planck Institute of Psychiatry, Munich, DE

61, Division of Research, Kaiser Permanente Northern California, Oakland, CA, US

62, Psychiatry & The Behavioral Sciences, University of Southern California, Los Angeles, CA, US

63, Department of Biomedical Informatics, Harvard Medical School, Boston, MA, US

64, Department of Medicine, Brigham and Women's Hospital, Boston, MA, US

65, Informatics Program, Boston Children's Hospital, Boston, MA, US

66, Wellcome Trust Centre for Human Genetics, University of Oxford, Oxford, GB

67, Department of Endocrinology at Herlev University Hospital, University of Copenhagen, Copenhagen, DK

68, Institute of Social and Preventive Medicine (IUMSP), University Hospital of Lausanne, Lausanne, VD, CH

69, Swiss Institute of Bioinformatics, Lausanne, VD, CH

70, Division of Psychiatry, Centre for Clinical Brain Sciences, University of Edinburgh, Edinburgh, GB

71, Mental Health, NHS 24, Glasgow, GB

72, Department of Psychiatry and Psychotherapy, University of Bonn, Bonn, DE

73, Statistics, University of Oxford, Oxford, GB

74, Psychiatry, Columbia University College of Physicians and Surgeons, New York, NY, US

75, School of Psychology and Counseling, Queensland University of Technology, Brisbane, QLD, AU

76, Child and Youth Mental Health Service, Children's Health Queensland Hospital and Health Service, South Brisbane, QLD, AU

77, Child Health Research Centre, University of Queensland, Brisbane, QLD, AU

78, Estonian Genome Center, University of Tartu, Tartu, EE

79, Medical Genetics, University of British Columbia, Vancouver, BC, CA

80, Statistics, University of British Columbia, Vancouver, BC, CA

81, DZHK (German Centre for Cardiovascular Research), Partner Site Greifswald, University Medicine, University Medicine Greifswald, Greifswald, Mecklenburg-Vorpommern, DE

82, Institute of Clinical Chemistry and Laboratory Medicine, University Medicine Greifswald, Greifswald, Mecklenburg-Vorpommern, DE

83, Institute of Health and Biomedical Innovation, Queensland University of Technology, Brisbane, QLD, AU

84, Humus, Reykjavik, IS

85, MRC Centre for Neuropsychiatric Genetics and Genomics, Cardiff University, Cardiff, GB

86, Virginia Institute for Psychiatric & Behavioral Genetics, Virginia Commonwealth University, Richmond, VA, US

87, Clinical Genetics, Vrije Universiteit Medical Center, Amsterdam, NL

88, Complex Trait Genetics, Vrije Universiteit Amsterdam, Amsterdam, NL

89, Solid Biosciences, Boston, MA, US

90, Department of Psychiatry, Washington University in Saint Louis School of Medicine, Saint Louis, MO, US

91, Department of Biochemistry and Molecular Biology II, Institute of Neurosciences, Center for Biomedical Research, University of Granada, Granada, ES

92, Department of Psychiatry, University of Groningen, University Medical Center Groningen, Groningen, NL

93, Department of Psychiatry and Psychotherapy, Medical Center of the University of Munich, Campus Innenstadt, Munich, DE

94, Institute of Psychiatric Phenomics and Genomics (IPPG), Medical Center of the University of Munich, Campus Innenstadt, Munich, DE

95, Division of Cancer Epidemiology and Genetics, National Cancer Institute, Bethesda, MD, US

96, Behavioral Health Services, Kaiser Permanente Washington, Seattle, WA, US

97, Faculty of Medicine, Department of Psychiatry, University of Iceland, Reykjavik, IS

98, School of Medicine and Dentistry, James Cook University, Townsville, QLD, AU

99, Institute of Health and Wellbeing, University of Glasgow, Glasgow, GB

100, deCODE Genetics / Amgen, Reykjavik, IS

101, College of Biomedical and Life Sciences, Cardiff University, Cardiff, GB

102, Institute of Epidemiology and Social Medicine, University of Münster, Münster, Nordrhein-Westfalen, DE

103, Institute for Community Medicine, University Medicine Greifswald, Greifswald, Mecklenburg-Vorpommern, DE

104, Department of Psychiatry, University of California, San Diego, San Diego, CA, US

105, KG Jebsen Centre for Psychosis Research, Norway Division of Mental Health and Addiction, Oslo University Hospital, Oslo, NO

106, Medical Genetics Section, CGEM, IGMM, University of Edinburgh, Edinburgh, GB

107, Clinical Neurosciences, University of Cambridge, Cambridge, GB

108, Internal Medicine, Erasmus MC, Rotterdam, Zuid-Holland, NL

109, Roche Pharmaceutical Research and Early Development, Neuroscience, Ophthalmology and Rare Diseases Discovery & Translational Medicine Area, Roche Innovation Center Basel, F. Hoffmann-La Roche Ltd, Basel, CH

110, Department of Psychiatry and Psychotherapy, University Medicine Greifswald, Greifswald, Mecklenburg-Vorpommern, DE

111, Department of Psychiatry, Leiden University Medical Center, Leiden, NL

112, Virginia Institute of Psychiatric & Behavioral Genetics, Virginia Commonwealth University, Richmond, VA, US

113, Computational Sciences Center of Emphasis, Pfizer Global Research and Development, Cambridge, MA, US

114, Institute for Molecular Bioscience; Queensland Brain Institute, The University of Queensland, Brisbane, QLD, AU

115, Department of Psychiatry, University of Münster, Münster, Nordrhein-Westfalen, DE

116, Institute of Medical Genetics and Pathology, University Hospital Basel, University of Basel, Basel, CH

117, Institute of Neuroscience and Medicine (INM-1), Research Center Juelich, Juelich, DE

118, Amsterdam Public Health Institute, Vrije Universiteit Medical Center, Amsterdam, NL

119, Centre for Integrative Biology, Università degli Studi di Trento, Trento, Trentino-Alto Adige, IT

120, Department of Psychiatry and Psychotherapy, Medical Center, University of Freiburg, Faculty of Medicine, University of Freiburg, Freiburg, DE

121, Psychiatry, Kaiser Permanente Northern California, San Francisco, CA, US

122, Medical Research Council Human Genetics Unit, Institute of Genetics and Molecular Medicine, University of Edinburgh, Edinburgh, GB

123, Department of Psychiatry, University of Toronto, Toronto, ON, CA

124, Centre for Addiction and Mental Health, Toronto, ON, CA

125, Division of Psychiatry, University College London, London, GB

126, Neuroscience Therapeutic Area, Janssen Research and Development, LLC, Titusville, NJ, US

127, Institute of Molecular and Cell Biology, University of Tartu, Tartu, EE

128, Psychosis Research Unit, Aarhus University Hospital, Risskov, Aarhus, DK

129, University of Liverpool, Liverpool, GB

130, Mental Health Center Copenhagen, Copenhagen Universtity Hospital, Copenhagen, DK

131, Human Genetics and Computational Biomedicine, Pfizer Global Research and Development, Groton, CT, US

132, Psychiatry, Harvard Medical School, Boston, MA, US

133, Psychiatry, University of Iowa, Iowa City, IA, US

134, Department of Psychiatry and Behavioral Sciences, Johns Hopkins University, Baltimore, MD, US

135, Department of Psychiatry and Psychotherapy, University Medical Center Göttingen, Goettingen, Niedersachsen, DE

136, Human Genetics Branch, NIMH Division of Intramural Research Programs, Bethesda, MD, US

137, Faculty of Medicine, University of Iceland, Reykjavik, IS

138, Child and Adolescent Psychiatry, Erasmus MC, Rotterdam, Zuid-Holland, NL

139, Psychiatry, Erasmus MC, Rotterdam, Zuid-Holland, NL

140, Psychiatry, Dalhousie University, Halifax, NS, CA

141, Division of Epidemiology, New York State Psychiatric Institute, New York, NY, US

142, Department of Clinical Medicine, University of Copenhagen, Copenhagen, DK
143, Department of Medical & Molecular Genetics, King's College London, London, GB
144, Psychiatry & Behavioral Sciences, Stanford University, Stanford, CA, US
145, NIHR BRC for Mental Health, King's College London, London, GB
146, Genetics, University of North Carolina at Chapel Hill, Chapel Hill, NC, US
147, Psychiatry, University of North Carolina at Chapel Hill, Chapel Hill, NC, US
